# On the salient limitations of the methods of assembly theory and their classification of molecular biosignatures

**DOI:** 10.1038/s41540-024-00403-y

**Published:** 2024-08-07

**Authors:** Abicumaran Uthamacumaran, Felipe S. Abrahão, Narsis A. Kiani, Hector Zenil

**Affiliations:** 1 Department of Physics and Psychology (Alumni), Concordia University, Montreal, Canada; 2 McGill University, McGill Genome Center, Majewski Lab, Montreal, Canada; 3 Centre for Logic, Epistemology and the History of Science, University of Campinas (UNICAMP), Campinas, Brazil; 4 DEXL, National Laboratory for Scientific Computing, Petrópolis, Brazil; 5 Department of Oncology-Pathology, Center for Molecular Medicine, Karolinska Institutet, Solna, Sweden; 6 Algorithmic Dynamics Lab, Karolinska Institutet, Solna, Sweden; 7 School of Biomedical Engineering and Imaging Sciences, King’s College London, London, UK

**Keywords:** Computational science, Information theory and computation, Complexity, Information theory

## Abstract

We demonstrate that the assembly pathway method underlying assembly theory (AT) is an encoding scheme widely used by popular statistical compression algorithms. We show that in all cases (synthetic or natural) AT performs similarly to other simple coding schemes and underperforms compared to system-related indexes based upon algorithmic probability that take into account statistical repetitions but also the likelihood of other computable patterns. Our results imply that the assembly index does not offer substantial improvements over existing methods, including traditional statistical ones, and imply that the separation between living and non-living compounds following these methods has been reported before.

## Introduction

The distinction between living and nonliving systems has long fascinated both scientists and philosophers. The question has been at the core of the areas of systems biology and complexity science since their inception, while the seminal concept of complexity—an irreducible emergent property among simpler components in a system—has long been believed to be central to the distinction between living systems and inanimate matter^1–5^.

The first to discuss this nexus of issues was Erwin Schrödinger, in his book “What is Life?”, exploring the physical aspect of life and cells, followed by Claude Shannon, whose concept of entropy, significantly shaped not only by communication theory but by his characterisation of life and intelligence, placed the concept of information at the core of the question about life. Shannon proposed that his digital theory of communication and information be applied to understanding information processing in biological systems^6^.

By solving not only the problem of a mathematical definition for randomness but also the apparent bias toward simplicity underlying formal theories, the concepts of algorithmic information, algorithmic randomness, and algorithmic probability from algorithmic information theory (AIT) abstract the issue away from statistics and human personal biases and choices to recast it in terms of fundamental mathematical first principles. These foundations are the underpinnings of coding methods, and they are ultimately what explain and justify their application as a generalisation of Shannon’s information theory. AIT has also been motivated by questions about randomness, complexity and structure in the real world, formulating concepts ranging from algorithmic probability^7^, that formalises the discussion related to how likely a computable process or object is to be produced by chance under information constraints, to the concept of logical depth^8^, that frames the discussion related to process memory, causal structure and how life can be characterised otherwise than in terms of randomness and simplicity.

A recently introduced approach termed “Assembly Theory” (AT), featuring a computable index, has been claimed to be a novel and superior approach to distinguishing living from non-living systems and gauging the complexity of molecular biosignatures with an assembly index or molecular assembly index (MA). In proposing MA as a new complexity measure that quantifies the minimal number of bond-forming steps needed to construct a molecule, the central claim is that molecules with high molecular assembly index (MA) values “are very unlikely to form abiotically, and the probability of abiotic formation goes down as MA increases”^9^. In other words, according to the authors, “high MA molecules cannot form in detectable abundance through random and unconstrained processes, implying that the existence of high MA molecules depends on additional constraints imposed on the process”^9^. We will use the notation “AT”, “assembly index”, or “MA” to refer to the aforementioned theory and the index derived therefrom.

The underlying intuition is that such an assembly index (by virtue of minimising the length of the path necessary for an extrinsic agent to assemble the object) would afford “a way to rank the relative complexity of objects made up of the same building units on the basis of the pathway, exploiting the combinatorial nature of these combinations”^10^.

In order to support their central claim, the authors of AT state that “MA tracks the specificity of a path through the combinatorially vast chemical space”^10^ and that, as presented in Marshall et al.^11^, it “leads to a measure of structural complexity that accounts for the structure of the object and how it could have been constructed, which is, in all cases, computable and unambiguous”.

## What a ZIP file can tell about life

The authors propose that molecules with high MA detected in contexts or samples generated by random processes, in which there are minimal (or no) biases in the formation of the objects, display a smaller frequency of occurrence in comparison to the frequency of occurrence of molecules in alternative configurations, where extrinsic agents or a set of biases (such as those brought into play by evolutionary processes) play a significant role.

However, we found that what the authors have called AT^9^ is a formulation that mirrors the working of previous coding algorithms—though no proper references or attributions are offered—in particular, statistical lossless compression algorithms, whose purpose is to find redundancies^12^. These algorithms were dictionary-based, like run-length encoding (RLE), Huffman^13^ and Lempev-Ziv (LZ)-based^14^. They were all launched early in the development of the field of compression for the purpose of detecting identical copies that could be reused.

Lossless compression, incorporating the basic ideas of LZ compression, has been widely applied in the context of living systems, including in a landmark paper published in 2005, where it was shown that it was not only capable of characterising DNA as a biosignature but also of reconstructing the main branches of an evolutionary phylogenetic tree from the compressibility ratio of mammalian mtDNA sequences^15^. The same LZ algorithms have been used for plagiarism detection, as measures of language distance, and for clustering and classification^15^. In genetics, it is widely known that similar species have similar nucleotide GC content and that, therefore, a simple Shannon Entropy approach on a uniform distribution of G and C nucleotides—effectively simply counting the exact repetitions of polymers^16^—can yield a phylogenetic tree. LZ compression has been used in this same context^17^, and is central to complexity applications to living organisms, which are based upon exactly the same grounds and on the idea of repetitive modules.

LZ77/LZ78 is at the core of AT, but its assembly index method is weaker than resource-bounded measures introduced before^18–20^. LZ-based schemes have been used in compression since 1977, and they are behind algorithms like zip, gzip, giff and others, exploited for the purposes of compression and as approximations to *algorithmic* (Solomonoff–Kolmogorov–Chaitin) *complexity*, which is one of the indexes from AIT. This is because compressibility is sufficient proof of non-randomness. Being one of the LZ compression schemes^12^, the assembly index calculation method looks for the largest substring matches, counting them only once as they can be reused to reproduce the original object. But it is weaker than other approximating measures because, by definition, it only takes into consideration identical copies rather than the full spectrum of causal operations to which an object may be subject (beyond simple identical copies).

Our results demonstrate that the claim that AT may help not only to distinguish life from non-life but also to identify non-terrestrial life, explain evolution and natural selection, and unify physics and biology is a major overstatement. (See also Supplementary Note 1, Supplementary Note 2 and Supplementary Results for a detailed presentation of the results). What AT amounts to is a re-purposing of some elementary algorithms in computer science in a sub-optimal application to life detection that has been suggested and undertaken before^8,21^, even generating the same results when applied to separating organic from non-organic chemical compounds^22^. By empirically demonstrating the higher predictive performance of AIT-based complexity measures, such as approximations to algorithmic complexity, to experimental applications in molecular classification, we extend the results reported before^22^ that had already—years before the introduction of AT—demonstrated the capabilities of these measures as regards separating chemical compounds by their particular properties, including organic from inorganic compounds. Further research based on the same underlying ideas of perturbation/mutation analysis together with algorithmic information theory has also been recently used to detect and decode bio- and technosignatures^23^.

## MA and compression algorithms

By employing different types of data (on the same subset of molecules^9,10^), as shown in Supplementary Figs. 2 and 3, we demonstrate that other measures applied to other (chemical and molecular) data reproduce what AT’s authors claimed was unique, though in fact it was not. We have shown that the same indexes used and shown in these figures, and reported to separate organic from non-organic compounds before^22^, also separate what the authors thought was a unique type of spectral data. Using exactly the same data input utilised by the authors of AT in their original paper^9^, we have shown that their MA index, also known as the assembly index, displays exactly the same behaviour as other complexity indexes. These results show that the assembly index calculation method not only is a compression scheme^12^, but also performs like one for all intents and purposes, and does not seem to afford any classificatory advantage either by virtue of its method or in combination with any property of the input data (e.g. mass spectra).

**Fig. 1:**
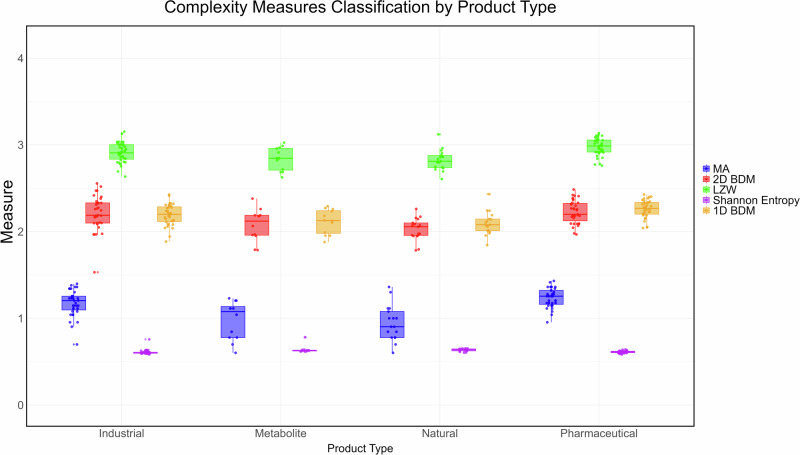
Classification of molecular complexity by multiple complexity indexes originally used to create the chemical space for the mass spectroscopy (MS) profiles (log-scale). A strong Pearson correlation with an *R*-value of 0.8823 was observed between 1D-BDM and MA for the 99 molecules available in the MS data set. LZW compression shared a close Pearson's correlation score of 0.8738 with MA. All correlation measures obtained a statistically significant one-tailed *p* value (*P* < 0.0001). All measures other than MA applied to bond molecular distance matrices, some of which outperform MA and mass spectra at distinguishing organic from non-organic molecules found in the MS dataset of the MA paper^9^, as demonstrated by greater separation and smaller variance results across the different complexity measures among the molecular subgroups. MA does not display any particular advantage when compared against proper control experiments and performs similarly to the simplest of the statistical algorithms applied to all the tested data representations, including molecular distance matrices (as shown here for all measures but MA) or the mass spectral data provided by the authors of Assembly Theory (shown on the plot from the authors' results that could not be fully reproduced due to lack of data made available^9^ but which we took at face value) for comparison purposes.

**Fig. 2:**
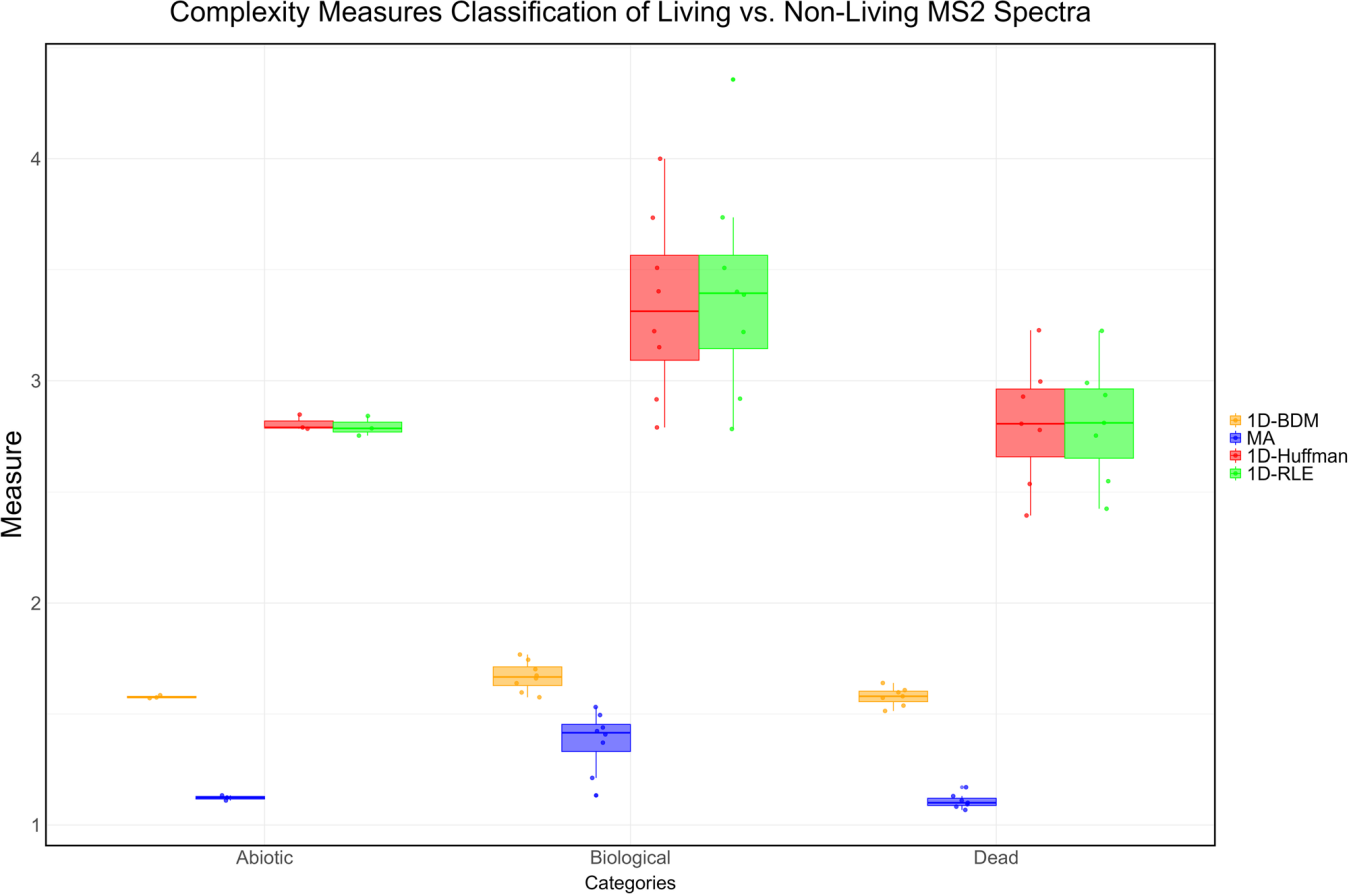
Analysis of organic versus non-organic molecules from mass spectral data by multiple complexity indexes: The strongest positive correlation was identified between MA and 1D-RLE coding (*R* = 0.9), which is one of the most basic coding schemes and among the most similar to the intended definition of MA, as being capable of ‘counting copies’ in 18 extracts for which the mass spectra was available. Other coding algorithms, including LZ and Huffman coding (*R* = 0.896), also show a strong positive correlation with MA. As seen, the compression values of both 1D-RLE and 1D-Huffman coding show overlapping and nearly identical medians (horizontal line at centre) and ranges on the whisker plot. The analysis further confirms our previous findings, with the similarity in performance in classifying living *vs*. non-living between MA and popular statistical compression measures (whose purpose is also to count identical statistical copies) leading us to make the case that MA is one (and the same as compression).

AT claims that MA can predict living *vs*. nonliving molecules, testing it against a small cherry-picked subset of biological extracts, between abiotic factors and inorganic (dead) matter. We repeated the experiment using the binarised MS2 spectra peaks matrices provided in the source data^9^. Our reproduced findings are shown in Figs. 1 and 2. (See also the Supplementary Results for more detailed information).

Thus, the coding indexes systematically outperform the MA index as a discriminant of living vs. non-living systems. MA works on the basis upon which all popular statistical lossless compression algorithms operate, the principle of ‘counting exact repetitions’ in data, which AT fully relies upon. These are basic coding schemes introduced at the inception of information theory and computer science that do not incorporate the many advances made in recent decades in the area of coding, compression and resource-bounded algorithmic complexity theory^24^ and cannot explain selection and evolution or unify physics and biology^25^ beyond the connections already made^26^.

As demonstrated here, the characterisation of molecules using mass spectrometry signatures is not a challenge for other equally computable and statistically-driven indexes. Other indexes are equally capable of discriminating biosignature categories, by InChI, by bond distance matrices or by mass spectra (MS2 peak matrices), thus disproving the claim that MA is the only experimentally valid measure of molecular complexity.

## Limitations of MA as a complexity measure

We have also shown that as soon as the MA index is confronted with more complicated cases of non-linear modularity, it underperforms or misses obvious regularities. As shown in this article and more detailed in Supplementary Note 2 and the Supplementary Results, our results show that MA, and its generalisation in the hypothesis called AT, is prone to false positives and fails both in theory and in practice to capture the notion of high-level causality beyond non-trivial statistical repetitions—that Shannon Entropy could not have already captured in the first place—which is necessary for distinguishing a serendipitous extrinsic agent (e.g. a chemical reaction resulting from biological processes) that constructs or generates the molecule of interest from a simple or randomly generated configuration (e.g. a chemical reaction resulting from environmental catalytic processes) or crystal-like minerals, as corroborated in Hazen et al.^27^.

The statistically significant separation of organic from non-organic compounds using molecular data and approximations to algorithmic complexity via compression, including using structural distance matrices empirically not very different from the mass spectral data used by AT, was first reported in Zenil et al.^22^. In another paper, we also made connections to selection and evolution, predating by several years^26^ a recent paper based on the same principles by the same group^25^, but unlike this paper, ours included tests on actual biological data, including but going beyond simple statistical repetitions (exact copies) using a Block Decomposition Method^20^. Very similar arguments and measures to those set forth by the authors of AT show, with actual generative examples, how modularity may emerge from simple mechanistic processes that follow algorithmic probability, explaining what AT meant to explain regarding how evolution may shortcut random processes towards building functional modular and hierarchical systems, and how evolution, drawing from a simplicity-biased distribution imposed by physical and chemical laws would, in a very fundamental fashion, lead to known evolutionary phenomena^26^.

The present article shows that the authors have failed to cite essential prior literature, mostly rehashing concepts and measures introduced before. The claims regarding the capabilities of AT—to characterise life, redefine time, find extraterrestrial life, explain selection and evolution, and unify biology and physics^25^—are shown to be unfounded or exaggerated, and if true, the same would be true of most of these other indexes.

In summary, while it is shown that AT is formally equivalent to a compression method (so that the assembly index calculation method is demonstrated to belong to the LZ family of compression schemes)^12^, here we have empirically shown that the best performance of molecular assembly does not outdistance other measures of a statistical nature (e.g. those based on Shannon Entropy) in any input data tested. Therefore, it conforms with the theoretical expectation and highlights a well-known mathematical property in data compression and complexity science: specifically, that different parsings (of an object) can perform equally in terms of compression rate. This directly reveals that the illustrative examples presented in later work^28^ fail to address our results, and further attempts^29,30^ seem to overlook the intrinsic deficiencies (both theoretical and empirical) in AT demonstrated in the present article.

Thus, we do not find AT to make deep or meaningful contributions to advancing the field, or to introducing new concepts, methods, novel applications or results that had not already been introduced or reported, especially in light of the hyperbolic claims associated with AT, and its multiple failures to cite the relevant literature. The limitations and drawbacks identified here extend to all applications of these methods^9–11,25^ and are based on their comparison to other weak statistical measures.

## Discussion: emergence and intrinsic complexity measures

Living systems are complex systems consisting of multiscale, multi-nested processes that are unlikely to be reducible to simplistic and intrinsic statistical properties such as those suggested by AT. Pure stochasticity is too strong an assumption and does not realistically represent the generative processes of molecules. Especially in the context of complex systems like living organisms, organic molecules may be the byproduct of intricate combinations of deterministic/computable and stochastic processes that govern the behaviour of the entire organism^2^^,20,31^ and its relationship with the way such agents exploit and interact with the information in their environments. In attempting to determine the living nature of an agent, any complexity measure that only looks at the agent’s internal structure, not taking into account an agent’s relationship with environmental state variables, is destined to fail.

We have shown that lacking the capability of detecting essential features of complex structure formation that go beyond a linear and combinatorial sequence space optimised for statistically identical repetitions, AT and its mathematical and computational methods based on decades-old coding schemes may return misleading values that would classify a low-complexity molecule as being extrinsically constructed by a much more complex agent, thus failing to appropriately characterise extraterrestrial life, contra the authors’ claim^9,25^. This extrinsic agent may be of a much simpler nature (e.g. a naturally occurring phenomenon). That is, in case a sufficiently complex environmental catalytic condition plays the role of this extrinsic factor (which increases the bias toward the construction of a more complex molecule), such a level of complexity would be completely missed by the capabilities of a simplistic measure such as MA, thereby rendering it prone to false positives. The presence of emergent properties that characterise the complexity of living systems cannot be reduced to a single paradigm or dimensionality, further confirming irreducibility as a hallmark of complex systems^5,31^.

### Supplementary information

Supplemental Information

## Data Availability

All the results, data and code are provided in our project GitHub repository: Mass spectrometry data is available in the Supplementary Information of Marshall et al.^9^. The Online Algorithmic Complexity Calculator (OACC) to reproduce the values of complexity indexes is available at http://www.complexity-calculator.com/. Text-to-binary conversion is available at https://www.rapidtables.com/convert/number/ascii-to-binary.html. The results of compression algorithms can be reproduced using https://planetcalc.com/9069/ for the Lempel-Ziv-Welch (LZW); https://www.dcode.fr/rle-compression for the run-length encoding (RLE); https://www.dcode.fr/huffman-tree-compression for the Huffman Coding.

## Code availability

Statistical correlation analysis was performed using GraphPad Prism v. 8.4.3., available at https://www.graphpad.com/scientific-software/prism/. Further computational tools to reproduce our results are described in the section “Data availability”.
