# Supplementary material for: On the salient limitations of the methods of assembly theory and their classification of molecular biosignatures
Source: NPJ Syst Biol Appl. 2024 Aug 7;10:82. doi: 10.1038/s41540-024-00403-y (PMC11306634; doi:10.1038/s41540-024-00403-y)
Supplement: Supplementary file 1 — Supplemental Information [file 41540_2024_403_MOESM1_ESM.pdf]

# Supplementary Information

Abicumaran Uthamacumaran[1,2], Felipe S. Abrahão[3,4], Narsis A. Kiani[5,6], and Hector Zenil* [6,7]

[1] Department of Physics and Psychology (Alumni), Concordia University, Canada.
[2] McGill University, McGill Genome Center, Majewski Lab, Canada.
[3] Centre for Logic, Epistemology and the History of Science, University of Campinas (UNICAMP), Brazil.
[4] DEXL, National Laboratory for Scientific Computing, Brazil.
[5] Department of Oncology-Pathology, Center for Molecular Medicine, Karolinska Institutet, Sweden.
[6] Algorithmic Dynamics Lab, Karolinska Institutet, Sweden.
[7] School of Biomedical Engineering and Imaging Sciences, King's College London, U.K..

*Corresponding author. Email: hector.zenil@cs.ox.ac.uk, hector.zenil@kcl.ac.uk

# 1 Supplementary Note 1

In recent papers, a method and measure have been proposed claiming to be capable of identifying and distinguishing molecules related to living systems versus non-living ones, among other capabilities.

Having identified a lack of control experiments and a limited analysis offered, we compared other measures of statistical and algorithmic nature that perform similarly, if not better, than the proposed assembly one at identifying molecular biosignatures.

Previous work claimed that the computable nature and tree-like structure of Assembly Theory (AT) was an advantage with respect to classifying the complexity of biosignatures. This is, however, one of its main weaknesses with respect to both grasping the complexity of the object and distinguishing it from a stochastically random ensemble.

We demonstrate that the assembly pathway method is a suboptimal restricted version of long-used compression algorithms and that MA performs similar to, if not worse, than other popular statistical compression algorithms. No strong compression or algorithmic complexity results is required to prove that AT and MA are ill-defined and underperform as compared to simple coding schemes. Our results show that despite the claims of experimental data, the assembly measure is driven mostly or only by InChI codes which had already been reported before to discriminate organic from inorganic compounds by other indexes.

Note that, depending on variations in application and context, the index measure featured in Assembly Theory has had several names, or can be referred to in different ways: pathway assembly (PA), object assembly (OA), or molecular assembly index (MA) as in [26, 27].

The concept of modularity of structure and recursive reconstruction from elementary building blocks has also been a feature long associated with life. In [21], for example, we proved that modularity can be built up from computation alone, and can therefore be characterised in a recursive fashion. However, the complexity of living systems also immediately suggests that simple measures, such as Huffman schemes or AT, are unable to characterise the complexity of life. Modularity also goes hand in hand with generative functions, particularly, of the recursive type. Characterisations in terms of thermodynamics [25, 40] have further enriched these measures, beyond statistical pattern recognition and number of steps, circling back to some original ideas related to what are believed to be the principles of living systems. Modularity, computability, and fundamental features of life have been richly intertwined and explored in tandem in the last century. Simple modular instructions can outperform the pathway assembly index because the latter

falls short to capture the subtleties of trivial modularity.

The group behind "Assembly Theory" are ignoring and neglecting decades of work in previous literature and resources such as work on resource-bounded complexity, self-assembly, modularity and self organization which is beyond the scope of this work. However, the challenges "Assembly Theory" is facing are what half a century of negative results in complexity theory have faced and (partially) solved by dealing with (semi) uncomputable measures after finding that computable measures, which fall into the trivial statistical ones, are of limited use, are ill-defined and can not only be highly misleading, but also a regression in the field.

We demonstrate in Section 2.2.1 that there are deceiving molecules whose low complexities arbitrarily diverge from the "random-like appearance" that the assembly pathway method assigns to them with arbitrarily high statistical significance. Our theoretical and empirical results imply that pathway assembly index is not an optimal complexity measure in general, and can return false positives.

In the main article, we have also suggested how the previous empirical methods can be applied to improved complexity measures that can better take advantage of the computational resources available.

## 1.1 Mischaracterisations

To understand the mathematical limitations underpinning AT, first note that the pathway assemblages are characterised by functions of the form

$$\begin{array}{cccc} g_k\colon & V(\Gamma)\times V(\Gamma) & \to & V(\Gamma) \\ & (z,x)=(z,(w_1,\dots,w_k,\dots)) & \mapsto & g_k(z,x)=(w_1,\dots,f(z,w_k),\dots) \end{array},$$

where $(w_1,\dots,w_k,\dots)$ denotes the object $x$ in the assembly space $(\Gamma,\phi)$ that results from the combination of other objects $w_1$, $w_2$, $\dots$, $w_k$, etc and function $f\colon V(\Gamma)\times V(\Gamma)\to V(\Gamma)$ gives the result of combining object $z$ with $w_k$. Being limited to joining operations—and this limitation becomes even more dramatic in the generative processes that we will discuss below—AT cannot deal with any variation of $x$ or $f$ beyond successive simple constructions. In the general case, most computable objects would be missed by statistical methods (like entropy and cognates such as AT). Since probability distribution uniformity does not guarantee randomness [9, 11, 42], most objects, both in theory and practice, cannot be recognised or characterised by weak computable measures, especially by those that are largely based on entropy measures such as statistical compression algorithms or AT.

Such a mischaracterisation has its roots in the reason any particular statistical test may fail to capture a mathematical formalisation of randomness, an

inadequacy which prompted the positing of algorithmic randomness [11, 17]. For every computable statistical test (e.g., obeying the law of large numbers or displaying Borel normality) for which there is a computably enumerable number of sequences that satisfy it, there are arbitrarily large initial segments of sequences that can be computed by a program, although these initial segments would be deemed random by statistical tests.

On the contrary, algorithmic randomness requires the sequence to be incompressible (and, as a consequence, uncomputable) across the board, or to pass *any* feasible statistical test. More formally, any sufficiently long initial segment of an algorithmically random infinite sequence is incompressible (except by a fixed constant) or, equivalently, the sequence does not belong to the infinite intersection of any Martin-Löf test [11, 17]. As a unidimensional example in the context of sequences, algorithmic complexity theorists very soon realised that an object such as $123456789101112\ldots$ could be very misleading in terms of complexity. Note that this sequence in fact defines the Champernowne constant $C_{10} = 0.123456789101112$, a complexity-deceiving phenomenon from the Borel normal numbers [42] that is generated by one of the most modular forms of a function type, recursion and iteration of a successor-type function $f(x_0, x_i) = x_i + x_0 = x_{i+1}$ for $x_0 = 1$. The ZK graph[42] which is constructed using the Champernowne constant as the degree sequence, was shown to be a near-maximal entropy graph with low algorithmic complexity [42]. The reader is invited to note how such a mathematical concept motivated the construction of deceiving molecules in Section 2.2.1.

In [15], the authors of Assembly Theory attempted to address what they thought were some concerns about an early preprint version of this manuscript. According to the authors, the statistical tools used were not appropriate, and the figures and comparisons above confound different types and sources of data with their application being unique in that it can be applied to data closer to what they think is the physical process, this is not correct. Regarding the statistical tools, even conceding (which we are not), that other statistical comparison methods are possible, it is not possible to manufacture desired results with one tool that with another one is found to be completely different. The authors seem to suggest that the greatest value of this paper is that some measures outperformed their Molecular Assembly (MA) index, and not that every other statistical index including some introduced in the 60s, applied to any chemical input data (not only mass spectra), reproduces and replicates what their paper [26] introduced as its main contribution.

## 1.2 On dictionary-based algorithms

The assembly method derived from the 'Assembly Theory' proposed by the original authors [28] consists roughly in finding a pattern-matching generative grammar behind a string by traversing and counting the number of copies needed to generate its modular redundancies, decomposing it into the statistically smallest collection of components that reproduce it without loss of information by finding repetitions that reproduce the object from its compressed form.

For purposes of illustration, let us take the example of ABRACADABRA, which the original authors have also used [28]. For molecular assembly (MA) to succeed it needs to have a discriminator and classifier able to characterise each repetition of $A$ and $N$ as the same, where $N$ is another character or some sub-unit of the structure with the same frequency as $A$ (e.g., a two-letter unit containing $A$, such as $AB$ or $RA$). In the ABRACADABRA example, MA deconstructs the sequence into unique blocks of five possible characters by adding a new character in subsequent steps, such that the minimal number of steps, considering only the frequency of the largest repeated block size (ABRA) just as it is done for LZ compression algorithms. The repeated binary or tertiary recursive structures (i.e., blocks of 2 or 3 letters) within the sequence, such as AB, RA, or BRA, are ignored in MA's minimal path search as in LZ.

The proposed Molecular Assembly (MA) [26], the assembly index, and the Assembly Theory in general [28] fall into the category of dictionary-based entropy encoding indexes and approaches and are indistinguishable from an implementation motivated by and based upon the principles of algorithmic complexity using LZ compression. In Section 1.2, we have shown the behavioural similarity between statistical compression and MA results consistent with the theoretical findings [6]. These popular statistical algorithms have been universally used for data compression and as computable estimations to algorithmic complexity [24] and logical depth [32, 39] including for classification purposes of living systems [13, 35, 39] of which AT is a special and, mathematically speaking, weak estimation.

Supplementary Figure 1 shows an illustration of the standard operation of Huffman coding in a typical example, compared to the principle advanced by the AT authors [28]. Proposed in the 50s, the Huffman coding exploits block redundancy by parsing objects, counting block recurrence and was one of the first, if not the first, dictionary-based coding algorithm [23].

As shown in Sup. Fig. 1 featuring the ABRACADABRA example, to the left (1A), we see the reconstruction of the sequence from a root node by the method proposed by AT, in general, and in this particular molecular applica-

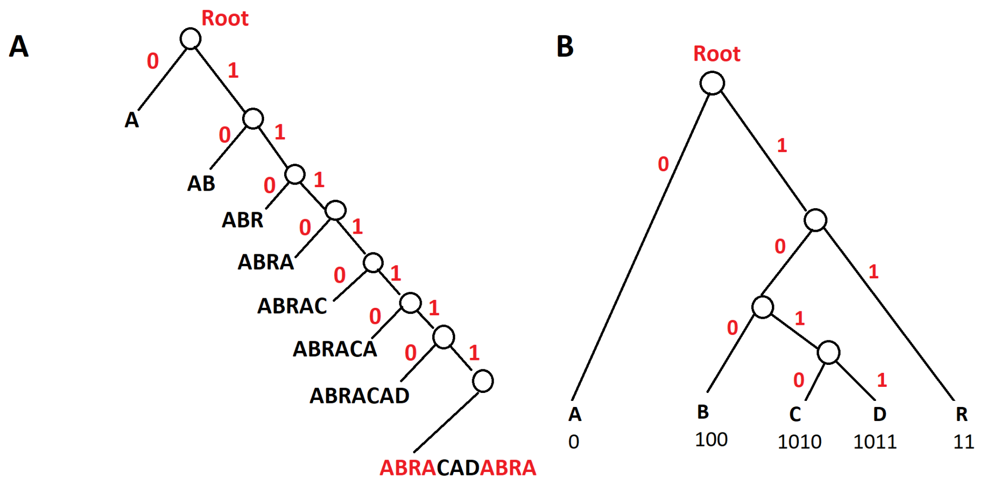


Supplementary Figure 1: ABRACADABRA tree diagrams for AT (A) and dynamic Huffman coding (B), both computable measures trivial to calculate. Huffman's was the first dictionary-based coding algorithm and is an optimal coding method able to characterise every statistical redundancy, including modularity, independent of such copy data representation [6].The (molecular) assembly index has been proven to be equivalent to LZ77/LZ78 [6]. In this example, Huffman's (which is also a sequential lossless compression algorithm that traverses strings from left to right) collapses the compression tree into a 4-level tree, while MA's is a 7-level tree. No natural evidence indicates that the assembly index (or MA) corresponds better to how nature works. However, the assembly index is identical to LZ compression [6]. In both cases, the resulting tree of this word problem characterises the same token and is able to reconstruct it in full, without any loss of information, by exploiting redundancy (identical copies) producing a set of possible cause-and-effect chains for which no empirical evidence exists in support of MA. Both LZ and Huffman, just as MA, converge to the same Shannon Entropy rate and can be used to guide a search in chemical space.

tion, when represented as a tree search diagram following binary branching rules. A bifurcation to the right denoted by 1 indicates a new assembly step, whereas a bifurcation to the left indicated by 0 from a node represents a fixed structure (block). The MA algorithm requires seven assembly steps to derive the sequence of interest. However, as shown to the left in Sup. Fig. 1, the Huffman coding tree optimises the sequence reconstruction by principles of recursivity in its search compression, as evidenced by the nested bifurcations.

Unlike algorithms like Huffman, MA lacks bifurcations in the assembly search and instead considers a combinatorial search space with a linear se-

quence progression that cannot be justified by causal chain progression because it may not have anything to do with how an object may have been assembled through many parallel processes. The authors, therefore, conflate causation with plausible assembly ordering.

Hence, it shirks the quantification of emergent hierarchical or nested structures (i.e., modularity optimisation) and intermediate structures within the sequence decomposition/compression. In contrast to MA, the recursiveness observed in complex molecules and biosignatures is detected by RLE and Huffman coding, and it does this in the most optimal way by providing the shortest tree algorithm (what the call 'assembly pathways') needed.

The results show that MA performs as any other coding/compression scheme because it is a compression scheme (even if they may have not intended to be so). The predictive power of MA, even if not unique or different, is therefore due to its information-theoretic properties based upon statistical compression of repeated patterns.

# 2 Supplementary Note 2

## 2.1 Methods

The list of MA values for all mass spectral signatures is available in [28]. Various complexity measures were used to classify living versus non-living molecules from the chemical space data in a four-category scheme: natural compounds, metabolites, pharmaceuticals, and industrial compounds, where the natural compounds include the amino acids, consistent with the classifications of Figure 2 in [28]. Further, the mass spectrometry (MS) data of the mixtures (biological extracts and non-living molecules) were categorized into three categories: abiotic, dead, and biological, as consistent with Figure 4 from [28]. The results were subjected to statistical analyses such as the Kolmogorov-Smirnov test, one sample t-tests, and Pearson correlation analysis using GraphPad Prism v. 8.4.3.

Mass spectrometry (MS) data of the extracts and the various molecules used to construct the chemical space for validating MA theory, were analyzed using various complexity measures, including the 1D-string and 2D-matrix Block Decomposition Method (BDM) [43, 44, 46], Shannon's entropy, and compression algorithms, including Lempel-Ziv-Welch (LZW), All data were first binarised using the online text-to-binary converter with ASCII / UTF-8 character encoding. Run Length Encoding (RLE), Huffman coding, and gzip. The InChI strings of the 99 molecules from (MW *vs.* MS data) of Fig. 2B, and the 114 molecules from Figure 3 (MS data standard curve) in [28] were binarised and analysed using the OACC (Online Algorithmic Complexity Calculator) app in R, which computed the 1D-BDM (block size of 2, alphabet size of 2, block overlap of zero) and Shannon Entropy scores. The LZW compression lengths were computed with an online LZW calculator using UTF-8 encoding for the 1D strings. Likewise, RLE and Huffman coding compression lengths were obtained using online calculators as additional lossless compression measures to assess the MS bio-signatures. The RLE calculator was set to character then count settings, while the Huffman coding calculator output was set to compression ratio. As for the Supplementary Figure 5, biological extracts (mixtures) analysis, we used the mass spectra peak matrices of the mixtures (MS2 peaks *vs.* the total number of peaks) for the above-discussed method/analysis, post-binarisation above the threshold.

For the unpaired (two-samples/independent measures) t-test with Welch's correction, at a degree of freedom (df) of 100, a critical t-value of 3.390 is expected for a two-tail P-value of 0.001 (i.e., 99.9% confidence). The t-value closest to 3.39 was found for the 1D-BDM and 2D-BDM [41, 44], with a

t-value of 6.410 and 6.561, respectively ($P < 0.0001$), both within the critical region of statistical significance. All complexity measures obtained a non-parametric Kolmogorov-Smirnov test value of $P < 0.0001$; the Kolmogorov-Smirnov distance D was smallest for the 1D-BDM and 2D-BDM, with both returning a value of 0.707.

Through these statistical assessments, the 1D-BDM and 2D-BDM at a binary conversion threshold of 3 were found to be robust discriminants of molecular complexity in classifying living *vs*. non-living molecules. The result is shown in Supplementary Figure 4.

To perform the 2D-BDM on the MS signatures (molecules), the structural distance matrix was extracted from the 2D-molecular structure SDF files for each molecule using the PubChem database. Binary conversion was performed on the matrices after flattening in R at five different conversion thresholds (i.e., -1, 0, 1, 3 and 5). The matrices were flattened by taking rows of values for the distance matrices, or pairs of values for the MS2 matrices (for Sup. Fig. 5) and ordering them by rows onto the Rapidtables ASCII/Unicode text string conversion calculator, and removing the spaces. The matrices can be simply pasted as a text, and converted to binary strings using the ASCII/UTF-8 character encoding and Space Output delimiter string settings. The binarised molecular distance matrices were processed by the PyBDM code to obtain the 2D-BDM scores for each molecule. Distance matrices at a binary conversion threshold of 3 were found to be optimal in differential analysis of the MA chemical space signatures and MS signatures into life *vs*. non-life categories. The matrices at a conversion threshold of 3 were used to compute the 2D-Huffman code and 2D-RLE compression lengths.

### 2.1.1 Description of Algorithmic Complexity Measures

The paper by Marshall et al. [26] introduced the molecular assembly (MA) algorithm as a new approach to assess the complexity of biochemical interactomes and distinguish biosignatures of life from abiotic chemistry. The hypothesis is that the molecular assembly (MA) algorithm models the assembly process of biomolecules.

To evaluate the MA algorithm's utility for analyzing complex biochemical systems, we benchmarked it against established methods from algorithmic information theory that quantify algorithmic complexity. Algorithmic information theory, rooted in computational complexity theory and Kolmogorov complexity, provides a rigorous mathematical framework to assess the information content of objects independent of the specific encoding. We applied several algorithmic information theory techniques to the same datasets ana-

lyzed in [26], including molecular distance matrices and mass spectrometry data of chemical interactomes. Our toolkit consisted of:

1D-BDM: The block decomposition method (BDM) segments data vectors into variable blocks and calculates the algorithmic complexity from the entropy of block sizes [44]. 1D-BDM analyzes one-dimensional data such as DNA sequences. 2D-BDM: The two-dimensional BDM acts on distance matrices and other 2D datasets. It divides matrices into rectangular blocks and computes complexity from block entropies.

LZW: The Lempel-Ziv-Welch (LZW) algorithm evaluates complexity by the number of steps needed to compress data (Ziv and Lempel, 1978). It builds a dictionary of sequences and measures complexity by dictionary size. Shannon Entropy: As a measure of uncertainty, or randomness, it quantifies information content based on symbol frequencies in data. Lower entropy indicates simpler patterns.

1D-RLE: Run-length encoding (RLE) [30] compresses data by counting repetitions of symbols. 1D-RLE counts runs in 1D sequences. 1D-Huffman: Huffman coding [23] compresses data by assigning shorter codes to more frequent symbols. 1D-Huffman operates on 1D sequences.

2D-RLE and 2D-Huffman: The 2D versions apply RLE and Huffman coding to 2D matrices by encoding runs or frequencies of matrix rows/columns.

Our results reveal fundamental limitations in using path lengths on assembly trees as a proxy for complexity. MA is most correlated to 1D-Huffman coding, which encodes more probable symbols with shorter codewords. Both exploit the frequency/probability distribution of components. However, this is just one facet of algorithmic complexity. Our toolkit provides a more complete view, with LZW, BDM, and 2D techniques assessing additional dimensions such as long-range sequence structure and 2D constraints. In summary, this paper benchmarks the novel MA algorithm against established algorithmic information measures for quantifying complexity in biochemical interactomes.

Our results reveal that MA has salient limitations as a standalone measure for biosignature complexity. The diverse measure toolkit provides a more robust perspective on assembly processes and algorithmic complexity in chemical systems. Our work elucidates the connections between MA and lossless compression, while demonstrating the power of algorithmic information measures that take into account regularities beyond trivial statistical redundancies.

### 2.1.2 PyBDM Code for CTM and BDM

The Coding Theorem (CTM) and Block Decomposition Methods (BDM) are resource-bounded computable methods [32, 36, 44] that attempt to approximate semi-computable measures that are a generalisation of statistical measures more powerful than the methods proposed in “Assembly Theory” as they combine global calculations of classical entropy with local estimations of algorithmic information content.

**Supplementary Algorithm 1** Python implementation of 2D-Block Decomposition Method (PyBDM)

```
import numpy as np
from pybdm import BDM
import pandas as pd
X = pd.read_csv(r'file directory',dtype=int)
bdm = BDM(ndim=2)
Z=X.to_numpy()
bdm.bdm(Z)
```

## 2.2 Expected false positives from complexity-deceiving molecules with arbitrarily high statistical significance

As we move beyond the realm of pure stochastic processes, complexity distortions become more problematic [3–5, 42]. As demonstrated in Theorem 2.4 and Corollary 2.5, there are (sufficiently large) deceiving molecules the complexities of whose respective generative processes arbitrarily diverge from the assembly index that the assembly pathway method assigns to them. We demonstrate in Section 2.2.1 that there are (sufficiently large) deceiving molecules the complexities of whose respective generative processes arbitrarily diverge from the assembly index that the assembly pathway method assigns to them. By a generative process [7] we mean any process that can be implemented, computed, or emulated by another equivalent (or identical) process so that it generates the pathway assembly and its object. In accordance with the assumptions and rationale in [26–28], those generative processes are exactly those composed of the assembling processes allowed (i.e., deemed physically possible by assembly theory’s chosen method) so that they in the end result in the constitution of molecules.

Notice that our results in Section 2.2.1 hold for any of such processes that belong to (abstract or physically implemented) computational classes much

weaker than that of Turing machines, e.g. resource-bounded Turing machine [8, 10, 11, 24, 32, 36] or simple forms of finite automata [29, 34]. Nevertheless, if the class of generative processes is constituted by processes that are also capable of universal computation, then Corollary 2.5 shows the complexity distortions (and, therefore, also the deceiving phenomena we explain below) can be equally bad or even worse. Therefore, AT fails to capture, both in theory (when resources can be unbounded) and in practice (when resources are limited), the minimality that is necessary for a complexity measure that may be claimed to be unambiguous and observer-independent.

While the calculation of MA may be prone to false negatives—due to partial fragmentation in energy collision analysis and the restriction to counting only valence rules in molecule synthesis (ignoring other chemical conditions)—this does not pose a challenge to the central claim made in [28]. Instead, MA aims at avoiding underestimation of the amount of molecules that result from random or abiotic processes, hence aiming at avoiding false positives. Thus, by directly tackling their central claims and this motivation, one demonstrates in Theorem 2.4 that MA in general fails to avoid false positives in the specific sense that it may not be able to distinguish a "complex" object that is in fact the outcome of randomly generated (resource-bounded) generative processes.

Under the same assumptions as in [27, 28], we construct in Theorem 2.4 a deceiving molecule that has a much larger MA value in comparison to the minimal information sufficient for a randomly generated generative process to single-handedly construct this molecule. Whatever arbitrarily chosen method is used to calculate the statistical significance level, the MA of this molecule is large enough to make the expected frequency of occurrence (estimated via the arbitrarily chosen AT) diverge from the actual probability. In this case, AT would consider such a molecule "biotic", resulting from extrinsic factors that increase biases toward certain pathways or that constrain the range of possible joining operations, although its sole underlying generative process in fact results from fair-coin-toss random events. This proven existence of false positives due to such a deceiving phenomenon is corroborated by our empirical findings, which show that MA displays a behaviour that is both structurally and empirically similar to traditional statistical compression methods. Indeed, the latter methods are already known to present distorted values [42], performing worse than more recent algorithmic-based methods [36]. Thus, they are prone to overestimating complexity, and consequently to presenting false positives for "high"-complexity objects which are in fact simple.

The key rationale behind this result is the computable nature of MA, so that given the set of biases and the joining operations allowed by the model,

objects with much higher MA can be constructed by much simpler (and, therefore, more probable) computable generative processes which can in turn be randomly generated. The more computationally cheap and tractable the AT's method is, the lower the complexity and the more limited the resources in which the deceiving phenomenon is expected to take place. Thus, in this context, MA (or any computable 'assembly' measure of this basic statistical type) will underestimate the frequency of occurrence of objects with high MA that in fact were constructed by much simpler randomly generated processes. This means that MA would misidentify molecules as byproducts (or constituents) of livings systems that resulted from evolutionary processes, while in fact these molecules might have been byproducts of single-handed computable systems that were randomly generated by a fair coin toss, and as such are not the result of an evolutionary process of optimisation over time.

Nevertheless, note that it is true that there are computable (lossless) encodings of a source, such as Huffman coding, that are proven to be optimal on average, but only if one knows a priori that the underlying processes generating the objects are purely stochastic (in particular, when one knows beforehand that the conditions of the source coding theorem are satisfied [14]). In this case, one can show that the minimum expected size of the encoded object converges to its expected algorithmic complexity [11, 14]. However, pure stochasticity is too strong an assumption, or does not realistically represent the generative processes of molecules.

On the one hand, living systems are complex systems consisting of multiscale, multi-nested processes that are unlikely to be reducible to simplistic and intrinsic statistical properties such as those suggested by AT. One cannot conceive a measure that only looks at the internal structure of an agent isolated from its environment and how it interacts with its external medium to determine its (non)living nature. Especially in the context of complex systems like living organisms, organic molecules may be the byproduct of intricate combinations or intertwinements of both deterministic/computable and stochastic processes that govern the behaviour of the entire organism and its environmental surroundings [33, 44].

On the other hand, in case a sufficiently complex (e.g., at the level of those we demonstrate in Section 2.2.1) environmental catalytic condition plays the role of the above extrinsinc factor (which increases the bias toward the construction of a more complex molecule), this level of complexity would be completely missed by the capabilities of simplistic measures such as MA, thereby rendering it as prone to false positives. More careful and deeper arguments regarding simplicity, recursivity, and the emergence of modularity in life have been advanced and are better grounded in a theoretical and methodological framework advanced in [21], where it was shown that exploiting first

principles of computability and complexity theories, modular properties in evolutionary systems may be explained.

As shown by Corollary 2.5, the deceiving phenomena can be equally bad or even worse in case the molecules are byproducts of complex systems that are somehow capable of universal computation. For example, in the case of advanced civilisations that are capable of artificially constructing living beings by computable processes, simplistic complexity measures such as MA can be intentionally misled with respect to what actually should be measured; or in the case of extrinsic environmental catalytic processes whose chemical dynamics' complexity are comparable to those of some randomly generated finite automata [34].

Our results suggest the presence in more realistic resource-limited scenarios of a property known to occur in theoretical complex systems science [7]: in the context of generative processes that are not purely stochastic, and are capable of displaying complexity or computation capabilities at the same level of those of the observers, there is no such thing as a generally optimal complexity measure that cannot be improved upon, since computable complexity measures are dependent on the observer (or the chosen formal theory). For example, without the necessary conditions being satisfied by the underlying stochastic process, one cannot generally guarantee such a convergence between the expected size of the encoded object and the expected algorithmic complexity that is assured by the source coding theorem.

### 2.2.1 Mathematical framework and assumptions

In order to demonstrate the presence of false positives in such realistic scenarios where both complexity and resources are limited (and, therefore, every process and measure is computable), one should account for the cases in which only a subclass of possible computable processes is allowed to perform the assembly rules (e.g., those that are allowed by the currently known law of physics in the case of molecules) in order to construct molecules. In this case, not every type of computable function may represent what is an effective or feasible process that constructs a molecule. Thus, in some cases the range of generative processes that can give rise to (or construct) a molecule may not comprise all possible computable functions. With this purpose, we employ a variation of the traditional algorithmic complexity and algorithmic probability studied in Algorithmic Information Theory (AIT). For this reason, we will employ a suboptimal form of the algorithmic complexity that is defined on non-universal programming languages (i.e., subrecursive classes).

Nevertheless, in an ideal (resource-*unbounded*) case in which the whole algorithm space indeed constitutes the set of all possible generative processes

for constructing the assembly space (e.g., when chemical processes are able to achieve the capability of effecting universal computation in the real world [33, 34]), we also show in Corollary 2.5 that the deceiving phenomenon hold in the same way (or can even be worse).

A deceiving phenomenon akin to the one employed in Theorem 2.4 can be found in [5] based upon the same AIT principles in [42], where sufficiently large datasets were constructed so that they deceive statistical machine learning methods into being able to find an optimal solution that in any event is considered global by the learning method of interest, although this optimal solution is in fact a simpler local optimum from which the more complex actual global optimum is unpredictable and diverges.

This phenomenon is also related to the optimality of the algorithmic complexity as an information content measure that takes into account the entire discrete space of computable measures [12, 18], or the maximality of the algorithmic probability as a probability semimeasure on the infinite discrete space of computably constructible objects, as demonstrated by the algorithmic coding theorem [11, 18, 24].

However, unlike in these previous cases, our proof is based on finding a deceiver algorithm that constructs an object with sufficiently high value of assembly index such that its expected frequency of occurrence is much lower than the algorithmic probability of the deceiver itself, and in this way passing the test of any statistical significance level the arbitrarily chosen formal theory may propose.

In order to achieve our results, we base our theorems on mathematical conditions that are consistent with the assumptions and results in [27, 28]. The first *assumption* that we specify with the purpose of studying a worst-case scenario is that the assembly space should be large enough so as to include those molecules (or objects) with sufficiently large MA (along with its associated sufficiently low pathway probability of spontaneous formation) relative to the algorithmic complexity of the deceiving program. For the sake of simplicity, we assume that the nested family $\mathcal{S}$ of all possible finite assembly spaces from the same basis (i.e., the root vertex that represents the set of all basic building blocks) is infinite computably enumerable. However, an alternative proof can be achieved just with the former—and more general—assumption that the assembly space may be finite but only needs to be sufficiently large in comparison to the deceiving program. Indeed, our assumption is in consonance with the authors' motivation (and/or assumption) that "biochemical systems appear to be able to generate almost infinite complexity because they have information decoding and encoding processes that drive networks of complex reactions to impose the numerous, highly specific constraints needed to ensure reliable synthesis" [28].

Closely related to the first assumption, we also *assume* that there always are molecules with arbitrarily low path probabilities, which follows from the notion that, as infinitesimal as it might be, there is always a chance of randomly combining elements from an unlikely (but possible) sequence of events so as to give rise to a certain complex molecule.

Thirdly, in accordance with the arguments in [26–28] that the computability and feasibility of their methods is an actual advantage in comparison with other complexity measures, here we likewise adopt these same *assumptions* so that the following are computable procedures:

- deciding whether or not a finite assembly space (or subspace) is well formed according to the joining operation rules that are allowed to happen;[1]

- calculating MA of a finite molecule (i.e., a finite object) in a well-formed assembly space (or subspace);[2]

- calculating the chosen approximation of MA (e.g., the split-branch version) of a finite molecule in a well-formed assembly space (or subspace);[3]

- calculating an upper bound for the pathway probability of spontaneous formation of a molecule in the denumerable nested family of possible finite assembly spaces;[4]

- calculating the significance level for a frequency of occurrence of a molecule in a sample so that this empirical probability distribution (i.e., the type of the sample) diverges from the pathway probability distribution of spontaneous formation of the molecule;[5]

### 2.2.2 Definitions and notation

Besides the notation from [27] for assembly theory, we also employ the usual notation for Turing machines and algorithmic complexity.

Respectively, as in [27, Definition 11] and [27, Definition 15], let $(\Gamma, \phi)$ denote either an *assembly space* or *assembly subspace*. From [27, Definition

[1]For example, one can employ the same criteria and allow the same rules established in [28].

[2]For example, as defined in [27, Definition 19].

[3]For example, as in [28, SI] and [27, Section 4.2].

[4]For example, this can be done by employing the methods developed in [27, SI] and [28]

[5]For example, by using a maximum-likelihood method or using the probability that a sample occurs with KL divergence larger than $\epsilon$ [14, 20].

19], we have that $c_\Gamma(x)$ denotes the *assembly index* of the object $x$ in the assembly space $\Gamma$.

Note that assembly spaces are finite. So, from our assumptions, we need to define a pathway assembly that can deal with arbitrarily large objects. To this end, let $\mathcal{S} = (\mathbf{\Gamma}, \mathbf{\Phi}, \mathcal{F})$ be an *infinite assembly space*, where every assembly space $\Gamma \in \mathbf{\Gamma}$ is finite, $\mathbf{\Phi}$ is the set of the correspondent edge-labeling maps $\phi_\Gamma$ of each $\Gamma$, and $\mathcal{F} = (f_1, \dots, f_n, \dots)$ is the infinite sequence of embeddings [22] (in which each embedding is also an *assembly map* as in [27, Definition 17]) that ends up generating $\mathcal{S}$. That is, each $f_i \colon \{\Gamma_i\} \subseteq \mathbf{\Gamma} \to \{\Gamma_{i+1}\} \subseteq \mathbf{\Gamma}$ is a particular type of assembly map that embeds a single assembly subspace into a larger assembly subspace so that the resulting sequence of nested assembly subspaces defines a total order $\preceq_{\mathcal{S}}$, where

$$(\Gamma_i, \phi_{\Gamma_i}) \preceq_{\mathcal{S}} \left(\Gamma_{i+1}, \phi_{\Gamma_{i+1}}\right) \text{ \textit{iff} } f_i(\Gamma_i) = \Gamma_{i+1} \ .$$

Let $\gamma = z \dots y$ denote an arbitrary path from $z \in B_{\mathcal{S}}$ to some $y \in V(\mathcal{S})$ in $\mathcal{S}$, where $B_{\mathcal{S}}$ is the basis (i.e., the finite set of basic building blocks) of $\mathcal{S}$ and $V(\mathcal{S})$ is the set of vertices of $\mathcal{S}$. Let $\gamma_x$ denote a rooted path from some $z \in B_{\mathcal{S}}$ to the object $x \in V(\mathcal{S})$.

Let ${\Gamma^*}_x$ denote a minimum rooted assembly subspace of $\Gamma$ from which the assembly index $c_\Gamma(x)$ calculates the augmented cardinality and that its longest rooted paths $\gamma_x$ ends in the arbitrary object $x \in V(\Gamma)$ as in [27, Definition 19].

As usual, let $\mathbf{U}$ be a universal Turing machine on a universal programming language $\mathbf{L}$. Let $\mathbf{U}(x)$ denote the output of the universal Turing machine $\mathbf{U}$ when $x \in \mathbf{L}$ is given as input in its tape. Let $\langle \cdot, \cdot \rangle$ denote an arbitrary recursive bijective pairing function [18, 24] so that the bit string $\langle x, y \rangle$ encodes the pair $(x, y)$, where $x, y \in \mathbb{N}$. Note that this notation can be recursively extended to $\langle \cdot, \dots, \cdot \rangle$ in order to represent the encoding of $n$-tuples.

We have that the (prefix) *algorithmic complexity*, denoted by $\mathbf{K}(x)$, is the length of the shortest prefix-free (or self-delimiting) program $x^* \in \mathbf{L}$ that outputs the encoded object $x$ in a universal prefix Turing machine $\mathbf{U}$, i.e., $\mathbf{U}(x^*) = x$ and the length $|x^*| = \mathbf{K}(x)$ of program $x^*$ is minimum. In addition, the *algorithmic coding theorem* [11, 12, 18, 24] guarantees that

$$\mathbf{K}(x) = -\log\left(\sum_{\mathbf{U}(p)=x} \frac{1}{2^{|p|}}\right) \pm \mathbf{O}(1) \ , \tag{1}$$

where $\sum\limits_{\mathbf{U}(p)=x} 2^{-|p|}$ is the *universal a priori probability* of $x$, which gives the probability of randomly generating (by an i.i.d. stochastic process) a prefix-free (or self-delimiting) program that outputs $x$. We also have it that $2^{-\mathbf{K}(x)}$

is called the *algorithmic probability* of $x$, which therefore converges to the universal a priori probability (except of an object-independent constant).

If the language $\mathbf{L}'$ is a proper subset of $\mathbf{L}$ such that language $\mathbf{L}'$ running on $\mathbf{U}$ is not able to decide every problem of Turing degree $\mathbf{0}$, then we have that $\mathbf{L}'$ is *not* a universal programming language, and the machine $\mathbf{U}$ defined upon language $\mathbf{L}'$ is a *Turing submachine* $\mathbf{U}/f$ [1], where $f$ is the partial function that computes the function $\mathbf{U}(x)$ for $x \in \mathbf{L}'$ as input. In other words, a Turing submachine is a Turing machine that can receive inputs in its tape (and possibly simulate other machines), but it is not universal. Weaker than Turing degree $\mathbf{0}$, a submachine can only compute problems in a subrecursive class of problems [1]. Thus, note that resource-bounded machines or total Turing machines are particular cases of Turing submachines [1].

As a consequence of the above definitions, we define the (prefix) *sub-algorithmic complexity*[6] $\mathbf{K}_f(x)$ to be the length of the shortest prefix-free (or self-delimiting) program $x^* \in \mathbf{L}'$ that outputs the encoded object $x$ when run on the Turing submachine $\mathbf{U}/f$ (i.e., $\mathbf{U}/f(x^*) = x$ and $|x^*|_f = \mathbf{K}_f(x) = \min\{|w| \mid \mathbf{U}(w) = x, \, w \in \mathbf{L}'\}$).[7] Thus, note that resource-bounded variants of the algorithmic complexity [10, 11, 24] are particular cases of sub-algorithmic complexity. Analogously, we will have that the *sub*-universal (a priori probability) distribution upon language $\mathbf{L}'$ is defined on the *sub*-universal a priori probability for each value $x$, which are given by

$$\sum_{\substack{\mathbf{U}(p) = x; \\ p \in \mathbf{L}'}} C \frac{1}{2^{|p|}} \, , \tag{2}$$

where $\mathbf{L}' \subseteq \mathbf{L}$ and $C \geq 1$ is a normalizing constant as in [2, Definition 3.6, Section 3.2.1] to ensure it is a probability measure and not a probability semimeasure. Note that, if $\mathbf{L}' = \mathbf{L}$, then one obtains the usual universal distribution instead of its subrecursive version.

Following these notions, we can now define the submachine that compute the allowed (physical, chemical, and/or biological) *generative processes* of an assembly space that assembly theory may arbitrarily choose. Following the same rationale from [26–28], those generative processes are exactly composed of the processes allowed by our current knowledge of physics so that they in the end result in the constitution of molecules.

As assumed to the above, and in accordance with the claims in [26–28], notice that the computability and feasibility of assembly theory's methods

[6]See also [1] where this terminology is also employed.

[7]If there is no program in $\mathbf{L}'$ that can output an object $x$, then one defines $\mathbf{K}_f(x) = \infty$.

directly implies the existence of such a submachine. Given any set of (physical, chemical, and/or biological) rules for assembling molecules that assembly theory arbitrarily chooses to be based on and that is consistent with our current knowledge of physics, one has it that for any particular pathway assembly resulting in a molecule, there is a correspondent program running on a submachine $\mathbf{U}_{\Gamma}$ for which one can apply the methods described in [26–28] to calculate the pathway probability and so on. This existence is trivially guaranteed to hold as long as the calculation of the assembly index is computable given an assembly space, which is already an assumed condition in [26–28]; moreover, any upper bound for the computational resources necessary to compute the assembly index straightforwardly implies the existence of an upper bound for the computational resources necessary for a program to run on $\mathbf{U}_{\Gamma}$ (i.e., submachine $\mathbf{U}_{\Gamma}$ is a type of resource-bounded Turing machine).

For the sake of simplifying notation, $\mathbf{U}_{\Gamma}$ denotes the Turing submachine $\mathbf{U}/f_{\Gamma}$. In this case, the function $f_{\Gamma}$ is the partial function that returns what $\mathbf{U}$ can compute with some $x \in \mathbf{L}_{\Gamma}$ as input, where $\mathbf{L}_{\Gamma} \subseteq \mathbf{L}$ is a (non-universal) programming language such that every allowed generative process—i.e., every generative process that is deemed physically possible by assembly theory's chosen method, in case the objects are molecules—of an assembly space is bijectively computed (or emulated) by a corresponding $\mathbf{U}(x)$. In other words, for every allowed generative process that can assemble objects into building another object, there is a program $x \in \mathbf{L}_{\Gamma}$ that computes (or emulates) this process. Conversely, for every $x \in \mathbf{L}_{\Gamma}$, one also has it that there is a corresponding generative process allowed by assembly theory, process which is computed (or emulated) by program $x \in \mathbf{L}_{\Gamma}$. Notice that in the forthcoming Theorem 2.4, we will show that if a certain generative process is equivalent (in terms of complexity and computation capability) to a particular type of algorithm running on this resource-bounded submachine, then the deceiving phenomenon takes places. Although our theoretical results in the next section prove the existence of false positives in the case this condition is met, our empirical results shown in Section 3 not only corroborate this prediction but in fact suggest this theoretical finding (or some weaker version of it) may be happening in much lower computational classes. This occurs because the assembly index either underperforms or does not outdistance other measures of a statistical nature, where distortions in complexity estimations are already well known to occur—which in particular is one of the reasons complexity science moved on from simplistic statistical measures and remains an evolving field of research.

In the case of infinite assembly spaces, one analogously defines language $\mathbf{L}_{\mathcal{S}} \subseteq \mathbf{L}$. (In the special case in which the generative process of the assembly

space $\mathcal{S}$ are capable of universal computation, then one has that $\mathbf{L}_{\mathcal{S}} = \mathbf{L}$ holds). Also for the sake of simplicity, let $\mathbf{K}_{\Gamma}$ denote the sub-algorithmic complexity $\mathbf{K}_{f_{\Gamma}}$. That is, $\mathbf{K}_{\Gamma}(x)$ gives the shortest program that can compute or emulate a generative process of the object $x$ in the assembly space $\Gamma$. In the case of infinite assembly spaces, one analogously defines the sub-algorithmic complexity $\mathbf{K}_{\mathcal{S}}$ and the sub-universal a priori probability upon language $\mathbf{L}_{\mathcal{S}}$.

### 2.2.3 Theoretical results

The main idea to achieve the following theoretical results is to construct a randomly generated program that receives a formal theory (which contains all the computable procedures and statistical criteria in assembly theory) as input. Then, it searches for a molecule (or object in an assembly space) with MA sufficiently high so as to make the pathway probability of spontaneous formation be sufficiently lower than the very own deceiving program's algorithmic probability, so that the divergence between these two probability distributions become statistically significant according to the arbitrarily chosen statistical method and significance level.

**Lemma 2.1.** *Let $\mathcal{S}$ be infinite computably enumerable. Let $\mathbf{F}$ be an arbitrary formal theory that contains assembly theory, including all the decidable procedures of the chosen method for calculating the assembly index (or approximating MA) of an object for a nested subspace of $\mathcal{S}$, and the program that decides whether or not the criteria for building the assembly spaces are met. Let $k \in \mathbb{N}$ be an arbitrarily large natural number. Then, there are a program $\mathrm{p}_y$, $\Gamma \subset \mathcal{S}$ and $y \in V(\Gamma)$ such that*

$$\mathbf{K}(y) + k \leq |\mathrm{p}_y| + k + \mathbf{O}(1) \leq c_{\Gamma}(y) \ , \tag{3}$$

*where the function $c_{\Gamma} \colon \Gamma \subset \mathcal{S} \to \mathbb{N}$ gives the MA of the object $y$ in the assembly space $\Gamma$ (or $\mathcal{S}$) and $\mathbf{U}(\mathrm{p}_y) = y$.*

*Proof.* Let $p$ be a bit string that represents an algorithm running on a prefix universal Turing machine $\mathbf{U}$ that receives $\mathbf{F}$ and $k$ as inputs. Then, it calculates $|p| + |\mathbf{F}| + \mathbf{O}(\log_2(k)) + k$ and enumerates $\mathcal{S}$ while calculating $c_{\Gamma}(x)$ of the object (or vertex) $x \in V(\Gamma) \subset V(\mathcal{S})$ at each step of this enumeration. Finally, the algorithm returns the first object $y \in V(\mathcal{S})$ for which

$$|p| + |\mathbf{F}| + \mathbf{O}(\log_2(k)) + k + \mathbf{O}(1) \leq c_{\Gamma}(y) \tag{4}$$

holds. In order to demonstrate that $p$ always halts, just note that $\mathcal{S}$ is infinite computably enumerable. Also, for any value of $c_{\Gamma'}(z)$ for some $z \in V(\Gamma') \subset V(\mathcal{S})$, there is only a finite number of minimum rooted assembly subspaces

(starting on any object in $B_{\mathcal{S}}$ and ending on $z$) whose augmented cardinality is $c_{\Gamma'}(z)$, where $B_{\mathcal{S}}$ is the basis (i.e., the finite set of basic building blocks [28]) of $\mathcal{S}$. This implies that there is an infinite number of distinct values of $c_{\Gamma'}(z)$. Now, let $\mathrm{p}_y = \langle k, \mathbf{F}, p \rangle$. Finally, from Equation 4 and basic properties in AIT, we have it that

$$\mathbf{K}(y) + k \leq |\mathrm{p}_y| + k + \mathbf{O}(1) \leq |p| + |\mathbf{F}| + \mathbf{O}(\log_2(k)) + k + \mathbf{O}(1) \leq c_{\Gamma}(y) \tag{5}$$

holds for some sufficiently large $k$. □

**Lemma 2.2.** *Let the conditions for Lemma 2.1 be satisfied. Let*

$$\mathbf{P} \colon \{\Gamma' | \exists x \left( c_{\Gamma}(y) = x \right), \Gamma' \subseteq \Gamma \subset \mathcal{S} \textit{ is rooted, and } y \in V(\Gamma')\} \to [0,1]$$

*be an arbitrary probability measure on the set of pathways in* $\mathcal{S}$ *and* $\mathrm{p}_{\mathbf{P}}$ *a program that computes a computable function that outputs an upper bound for* $\mathbf{P}$ *such that for every* $\epsilon' > 0$ *and* $\Gamma \subset \mathcal{S}$*, there are* $\Gamma' \subset \mathcal{S}$ *and* $x \in V(\Gamma')$ *with* $\Gamma \subseteq \Gamma'$ *and* $\mathbf{P}({\Gamma^*}_x) \leq \mathbf{U}(\langle {\Gamma^*}_x, \mathrm{p}_{\mathbf{P}} \rangle) < \epsilon'$. *Let* $1 \geq \epsilon > 0$ *be an arbitrary encodable real number. Let* $k \in \mathbb{N}$ *be an arbitrarily large natural number. Then, there are a program* $\mathrm{p}_\epsilon$*,* $\Gamma \subset \mathcal{S}$*, and* $y \in V(\Gamma)$ *such that Lemma 2.1 is satisfied with* $y$ *and*

$$\mathbf{K}(y) + k \leq |\mathrm{p}_\epsilon| + k + \mathbf{O}(1) \leq c_{\Gamma}(y) \ , \tag{6}$$

*and*

$$\mathbf{P}({\Gamma^*}_y) \leq \mathbf{U}(\langle {\Gamma^*}_y, \mathrm{p}_{\mathbf{P}} \rangle) < \epsilon \tag{7}$$

*hold, where* $\mathbf{U}(\mathrm{p}_\epsilon) = {\Gamma^*}_y$.

*Proof.* Let $p'$ be a bit string that represents an algorithm running on a prefix universal Turing machine $\mathbf{U}$ that receives $\mathrm{p}_{\mathbf{P}}$, $\epsilon$ and $k$ as inputs. Then, it enumerates the assembly pathways ${\Gamma^*}_x$ in $\mathcal{S}$ such that $|p| + |\mathbf{F}| + \mathbf{O}(\log_2(k)) + |p'| + |\mathrm{p}_{\mathbf{P}}| + \mathbf{O}(\log_2(\epsilon)) + k + \mathbf{O}(1) \leq c_{\Gamma}(x)$, $\mathbf{U}(\langle {\Gamma^*}_x, \mathrm{p}_{\mathbf{P}} \rangle) < \epsilon$, and Lemma 2.1 holds for $x$ given $k$. Finally, it returns this first ${\Gamma^*}_x$ in this enumeration. Now, let $\mathrm{p}_\epsilon = \langle \mathrm{p}_{\mathbf{P}}, \epsilon, k, p' \rangle$. Therefore, in addition to the arguments in the proof of Lemma 2.1, the desired theorem follows from the fact that program $p'$ always halts because of our initial assumptions on program $\mathrm{p}_{\mathbf{P}}$ and the probability distribution given by $\mathbf{P}$. □

**Lemma 2.3.** *Let the conditions for Lemmas 2.1 and 2.2 be satisfied. Let* $\mathbf{F}' \supseteq \mathbf{F}$ *be a formal theory that also includes the chosen statistical method, the criteria for the arbitrarily chosen statistical significance level, the chosen computable method for approximating* $\mathbf{P}$ *from above with program* $\mathrm{p}_{\mathbf{P}}$*, and the acceptable maximum error* $\mathscr{E} \in \mathbb{N}$ *for an overestimation of the complexity of*

*an object in $\mathcal{S}$. Then, there are a program $\mathrm{p}_d$, $\Gamma \subset \mathcal{S}$, and $y \in V(\Gamma)$ such that Lemma 2.2 is satisfied with $y$ and $\mathbf{F}'$ decides that the divergence of the (sub-)universal distribution from $\mathbf{P}$ is statistically significant, where $\mathbf{U}(\mathrm{p}_d) = \Gamma^*{}_y$ and $|\mathrm{p}_d| + \mathscr{E} < c_\Gamma(y)$ hold.*

*Proof.* Let $\mathrm{p}_d$ be a bit string that represents an algorithm running on a prefix universal Turing machine $\mathbf{U}$ that includes the computation of $\mathrm{p}_k$ and $\mathrm{p}_\epsilon$ (which are programs defined in the proofs of Lemmas 2.1 and 2.2) as subroutines. Then, it searches for the first $\Gamma^*{}_x$, sufficiently small value of $\epsilon$, and sufficiently large value of $k \geq \mathscr{E} + \mathbf{O}(1)$ such that Lemmas 2.1 and 2.2 are satisfied with $x$, $|\mathrm{p}_d| + \mathscr{E} < |p| + |\mathbf{F}'| + \mathbf{O}(\log_2(k)) + |p'| + |\mathrm{p}_\mathbf{P}| + \mathbf{O}(\log_2(\epsilon)) + k + \mathbf{O}(1) < c_\Gamma(x)$ holds, and the divergence of $2^{-|\mathrm{p}_d|}$ from $\mathbf{U}(\langle \Gamma^*{}_x, \mathrm{p}_\mathbf{P} \rangle)$ is statistically significant according to the formal theory $\mathbf{F}'$. Finally, the algorithm returns this first assembly pathway $\Gamma^*{}_x$ as output. Note that, since the value of $2^{-|\mathrm{p}_d|}$ is fixed, one can always employ program $\mathrm{p}_\mathbf{P}$ and the statistical criteria in theory $\mathbf{F}'$ to find an arbitrarily lower probability than $2^{-|\mathrm{p}_d|}$ so that the resulting probability distribution (i.e., the probability distribution given by $\mathbf{P}$) diverges from the sub-universal (a priori probability) distribution. This holds because: of the algorithmic coding theorem, which implies that $2^{-|\mathrm{p}_d|}$ is a lower bound for the sub-universal a priori probability upon language $\mathbf{L}'$, where $\mathrm{p}_d \in \mathbf{L}' \subseteq \mathbf{L}$; and of the fact that $\mathrm{p}_\mathbf{P}$ is a program that computes a (computable) function that outputs an upper bound for $\mathbf{P}$.[8] Additionally, this divergence eventually becomes statistically significant (as the divergence increases) because the probability of occurrence of a sequence of events following an empirical probability distribution, which diverges from the original distribution that the events are generated, eventually decreases as the divergence sufficiently increases. Also note that $|\mathrm{p}_d| \leq |\mathrm{p}_k| + |\mathrm{p}_\epsilon| + \mathbf{O}(1)$. Therefore, since $k$ and $\epsilon$ were arbitrary in Lemma 2.2 and all the statistical methods in $\mathbf{F}'$ are decidable by assumption, we have that $\mathrm{p}_d$ always halts. □

**Theorem 2.4.** *Let the conditions for Lemma 2.3 be satisfied. Let $\mathcal{S}$ be an infinite assembly space whose set of randomly generated (computable) generative processes include one that can effect at least the computation of program $\mathrm{p}_d$, where Lemma 2.3 holds for $\mathrm{p}_d$ and $y \in V(\mathcal{S})$. Then:*

- *the complexity error is larger than $\mathscr{E}$ (except for an independent constant);*

[8] Also note that $\mathbf{F}'$ does not actually need to be able to compute the value of the sub-universal a priori probability of $x$ because one already knows $2^{-|\mathrm{p}_d|}$ is a lower bound for it and $\mathbf{U}(\langle \Gamma^*{}_x, \mathrm{p}_\mathbf{P} \rangle)$ is an upper bound for the *optimal* pathway probability of $x$.

- *and this error implies a statistically significant (according to* $\mathbf{F}'$*) distinct frequency of occurrence of* $y$ *than it was expected from the chosen assembly theory.*

*Proof.* From Lemma 2.3, we have it that $\mathbf{U}\left(\mathrm{p}_d\right) = \Gamma^*{}_y$. Thus, from our assumptions and the definition of $\mathbf{K}_{\mathcal{S}}$, we have it that $\mathbf{K}_{\mathcal{S}}\left(y\right) \leq |\mathrm{p}_d| + \mathbf{O}(1)$, which proves that the complexity error is larger than $\mathscr{E}$ from Lemma 2.3. We also have that the probability of an assembly pathway being constructed by a randomly generated computable process is given by the sub-universal a priori probability of $\Gamma^*{}_y$ upon language $\mathbf{L}_{\mathcal{S}}$, i.e.,

$$\sum_{\substack{\mathbf{U}\left(p\right) = \Gamma^*{}_y; \\ p \in \mathbf{L}_{\mathcal{S}}}} C \, \frac{1}{2^{|p|}} \ . \tag{8}$$

Therefore, by replacing $\mathbf{L}'$ with $\mathbf{L}_{\mathcal{S}}$ in the proof of Lemma 2.3, we achieve a statistically significant (according to $\mathbf{F}'$) distinct frequency of occurrence of $y$ than it was expected from the chosen assembly theory. □

**Corollary 2.5.** *Let the conditions for Lemma 2.3 be satisfied. Let* $\mathcal{S}$ *be an infinite assembly space whose randomly generated (computable) generative processes are capable of universal computation. Then:*

- *the complexity error is larger than* $\mathscr{E}$ *(except for an independent constant);*

- *and this error implies a statistically significant (according to* $\mathbf{F}'$*) distinct frequency of occurrence of* $y$ *than it was expected from the chosen assembly theory.*

*Proof.* The proof follows directly from the fact that $\mathbf{K}\left(y\right) \leq \mathbf{K}_f\left(y\right) + \mathbf{O}(1)$ and from replacing $\mathbf{L}'$ with $\mathbf{L}$ in the proof of Lemma 2.3. □

# 3 Supplementary Results

In 2017 [35], we explored the question of molecular and chemical separation using several data inputs and methods were we founded we could classify correctly organic from inorganic compounds using a standard chemical database of more than 15,000 compounds (as opposed to only 100 as in the Assembly Theory paper [28].) We were able to successfully separate organic from inorganic compounds using not only chemical nomenclature names (InChI) but also structural bond network distance matrices found in the ChemicalData (PubChem) repository available in Wolfram Mathematica.

In the early 2010s, we introduced a measure called Block Decomposition Method of which we proved most of its features in [43] demonstrating that in the worse case without updating the underlying (universal) distribution based on the principles of algorithmic complexity, it would converge to Shannon Entropy but in the average case it would combine the power of what we called the Coding Theorem Method [16] with traditional information theory calculating local estimations of algorithmic complexity to find local patches of causality. The Block Decomposition Method looks for identical repeated blocks counting their abundance but also takes into account blocks that may have been assembled/produced by the same generating underlying mechanism, hence taking the concept of abundance of patterns beyond trivial statistical repetitions. In multiple applications, we connected it to molecular and chemical complexity [35], behavioural complexity [19, 38], cell biology [44], molecular biology [37], causality [45–47] and selection and evolution [21].

Despite their statistical limitations, RLE, LZ and Huffman's coding algorithms are among the simplest coding algorithms introduced in the 1960s and 1970s. They are known not to be optimal for statistical or algorithmic compression, but optimal at doing what they were intended to do, that is, counting statistical copies in the form of minimum code lengths and approaching Shannon Entropy rate in the limit.

## 3.1 Molecular Assembly classification exhibits similar or lower performance than existing statistical algorithms on multiple data types

We compared the performance of 'Molecular Assembly' (MA) with measures of statistical (statistical compression) and algorithmic (BDM) nature under the four mass spectroscopy (MS) categories seen in [28, Figures 2-4]. Note, that mass spectroscopy can also be referred to as mass spectrometry, and is

used interchangeably.

Various measures such as BDM, LZW, and RLE were first computed on the InChI code strings to show that other data inputs were able to classify the same molecules/compounds in [26], followed by an application to bond connectivity matrices of structural chemical networks. Finally, we applied all these indexes to the 2D mass spectra matrices of the molecules or chemical systems in [28, Figures 2-4]. To this end, we binarised the 2D mass spectrometry matrices provided in their supplementary data according to our methodology as described in Section 2.1, and computed the rest of the values for all other measures. We then used correlation analysis and a combination of simple statistical tests to compare MA to the other indexes in classifying the chemical systems and molecules from Marshall's results in [28, Figures 1-4], as life *vs*. non-life categories. The T- test and Kolmogorov-Smirnov (KS) test have been used for this purpose. Unlike the t-test statistic, the Kolmogorov-Smirnov test provides a non-parametric goodness-of-fit test, assuming the data does not come from a Gaussian (Normal) distribution. It should be noted that while KS-tests by themselves are not classification algorithms, they are useful as part of a classification pipeline by providing a statistical measure of the similarity between sample distributions. A combination of the KS-test p-value along with other metrics like corrected t-test p-values and R-squared values from the correlation analysis allows for a more determination of whether two distributions are statistically distinguishable, and to evaluate the categories used in the figures in [28]. While other types of statistical tests are possible, in general it is not the case that one test will contradict other. Even conceding that these other measures do not necessarily outperform Molecular Assembly (MA) or the assembly index (which according to our statistics they do), this work still suggests all these other measures produce the same results and should have been explored in a comparison analysis in the original study [26] for good scientific practice. Had it been done, the authors may have learned more about their own index and may have placed it in the right context, cite previous literature, and provide a proportional coverage of its limited impact given its incremental, if anything, contribution.

Supplementary Figure 1 shows an illustration of the standard operation of Huffman coding as the first dictionary-based coding scheme on a typical example next to a popular example used by the authors of Assembly Theory [28]. In Supplementary Figures 2 and 4, we test a set of statistical measures in preparation for the incorporation of mass spectroscopy data as done in [26]. Both 1D-RLE and 1D-Huffman coding schemes show a strong statistical correlation and linear correspondence with MA (see Sup. Fig. 2). The one-dimensional and simplest lossless compression algorithms RLE and

Huffman code compression lengths showed the strongest Pearson correlations with MA at R-values of 0.9001 and 0.896. The complete correlation analysis of the 114 molecules classification is provided in Supplementary Table 3.

The results do not come as a surprise because these algorithms count repetitions even if they may do so in different ways or may represent data in a different fashion they still are able to pick the same repetitive signal independent of representation guaranteed by the principles of information theory as one cannot create or erase patterns by simply changing the tokens or the underlying vocabulary from a direct translation. All these results conform with the theoretical expectation of MA to be a compression scheme of the LZ family as proven in [6].

It has long been established that prediction is equivalent to lossless coding/compression and vice versa [18]. The compressed version of a phenomenon is a model that has to abstract its most salient properties. These results imply that these are not abstract disconnected properties of data but the properties that are most often the best and right explanation of a process. This is the main assumption of science. Therefore, compression is not only for data compression but is at the core of the practice of science, simulation, modelling, abstraction and prediction. Yet, the misunderstandings surrounding compression, Shannon Entropy, Kolmogorov complexity, Turing machines, computation universality, computability, evolutionary and development biology is deep and manifold [26–28]

## 3.2 Molecular classification by structural information in nomenclature codes

Here we show how different data types classify the data in the same way as Molecular Assembly does, without access to other type of data or other algorithms different from statistical or compression/coding schemes as reported in [35] before moving to molecular bond distance matrices also reported in [35] and mass spectral data as used in [26].

InChI is an open standard identifier for chemical databases that facilitates effective identification of chemical compounds. The InChI algorithm converts input structural information into a unique identifier in a three-step process. A normalisation (to remove redundant information), canonicalisation (to generate a unique number label for each atom), and serialisation (to assign a string of characters) and as such, InChI codes include all the necessary information to uniquely map and build the structure of every chemical compound.

The application of traditional statistical indexes analysis reveals the same

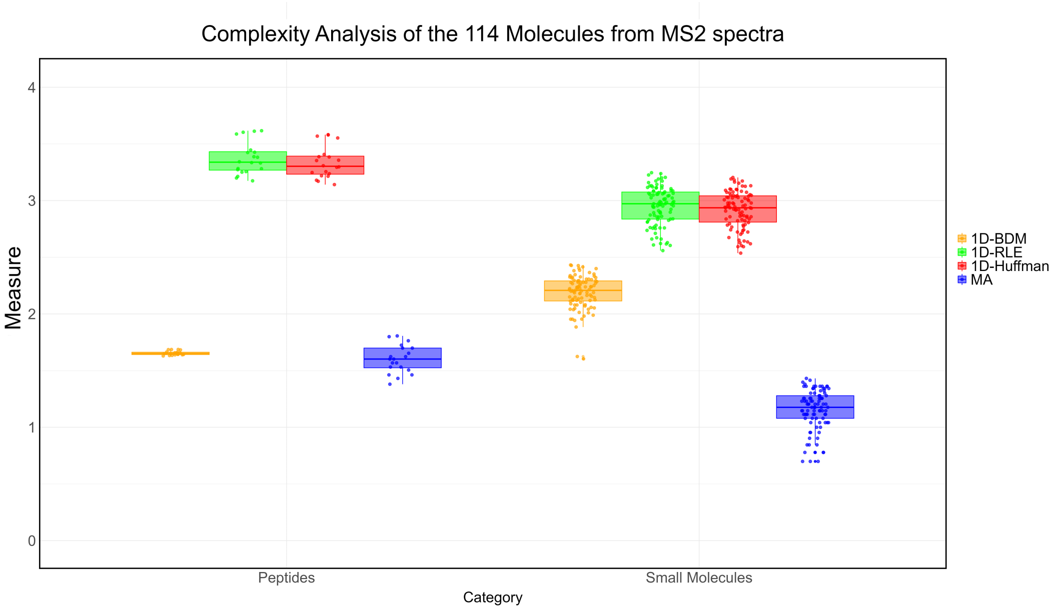


Supplementary Figure 2: Correlation plot between the 'Molecular Assembly' (MA) index (taken at face value, not recalculated only reclassified according to a larger category space as in [35]) and other compression scores on InChI codes as performed in [35] with the molecules in [26]. The vertical axes are the five complexity scores in log normalised scale for comparison purposes.

separation as the reported in MA that also failed to cite the previous results in the field [35]. These popular statistical lossless compression algorithms are based on the same counting-copies principles used by Assembly Theory [28] and the Molecular Assembly [26].

As seen in Sup. Fig. 2, the values of both 1D-RLE and 1D-Huffman coding show overlapping and nearly identical medians (horizontal line at centre) and ranges on the whisker plot. The plot shows that while the input data (mass spectral for MA versus InChI nomenclature for others), in all cases, a significant different signal across categories is found allowing each class to have different statistics for proper classification, in particular among natural and organic or inorganic.

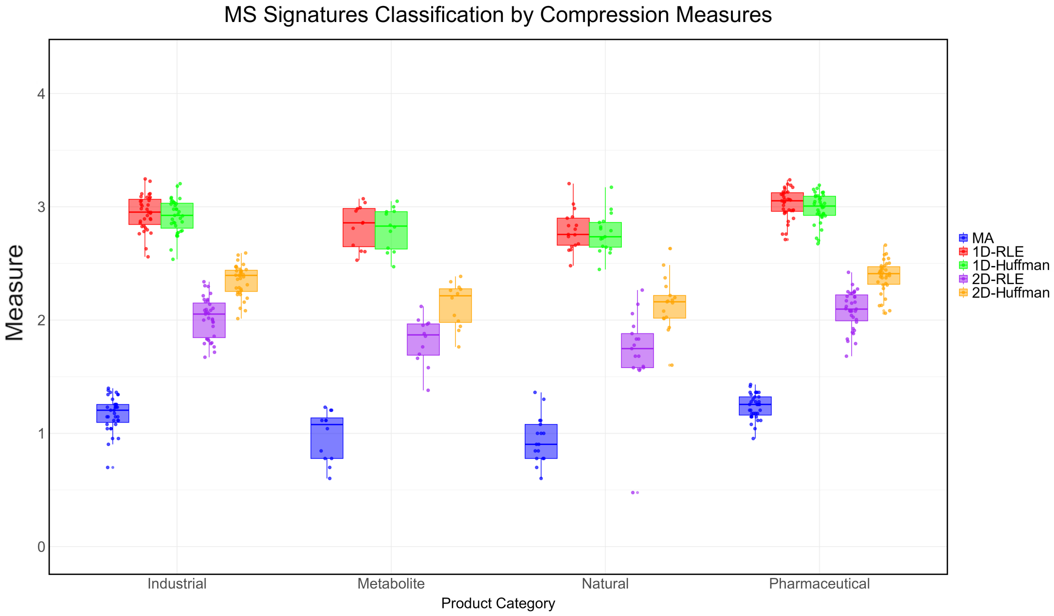


Supplementary Figure 3: Same analysis of the application of multiple statistical indexes on the same set but according to the categories in [28, Figure 3] showing the same separating properties. The strongest Pearson correlation was identified between 1D-BDM and the category of molecules ($R= 0.828$; $P<0.0001$).

The comparison of measures across the four categories of MS molecules is shown in Supplementary Table 2 with respect to increasing the molecular weight (MW) to better visualise the trends across living and non-living bio-signatures. The Pearson correlation test was assessed on the various complexity and compression measures in relation to molecular weight (MW) with an alpha value of 0.01 (99 percent confidence interval) for which the one-tailed

| Statistic | 1D-BDM | 1D-RLE | 1D-Huffman | MA |
|---|---|---|---|---|
| Pearson Correlation (R) | 0.828 | 0.704 | 0.713 | 0.7111 |
| 99% confidence interval | 0.87-0.76 | 0.59-0.78 | 0.61-0.793 | 0.60-0.79 |
| R squared | 0.686 | 0.495 | 0.509 | 0.506 |

Supplementary Table 1: Table of Pearson Correlation values of MA and complexity indices across the two categories (small molecules and peptides) of the molecules in [28, Figure 3]. BDM, RLE, and Huffman are given in log-normalised bits. As shown, BDM generates better statistics than MA without any adaptions or modifications, while Huffman shows near identical correlation performance as MA, thereby, supporting the findings.

p-values were significant (P<0.0001) for all indexes compared in Sup. Table 2. The one-tail P-value tests were performed instead of the two-tail tests since our (previous) analyses inferred a unidirectional linear relationship in the trend patterns. As shown in Sup. Table 2, 1D-BDM had the highest Pearson correlation with MW (R=0.905), followed by LZW compression (R=0.9028). MA has a correlation score of 0.8055.

### 3.3 Molecular classification by chemical bond distance matrices and spectral data

We now test these measures on objects closer to the instrument measurement of the chemical data, including the mass spectral data used by Marshall et al. in [26]. The two-dimensional distance matrices of the mass spectroscopy (MS) data were binarised and converted using a threshold of three before being subjected to the compression algorithms. The 2D-RLE and 2D-Huffman code compression lengths obtained Spearman correlation values of 0.7967 and 0.7537, respectively with MA (the Pearson scores were comparable). The gzip compression showed a Spearman correlation of 0.804.

A strong Pearson correlation with an R-value of 0.8823 was observed between 1D-BDM and MA for the 99 molecules available in the MS data set (see Sup. Fig. 4). LZW compression shared a close Pearson's correlation score of 0.8738 with MA. All correlation measures obtained a statistically significant one-tailed p-value ($P < 0.0001$).

The Molecular Assembly indices did not show any significant advantage when compared with other measures that were introduced several decades

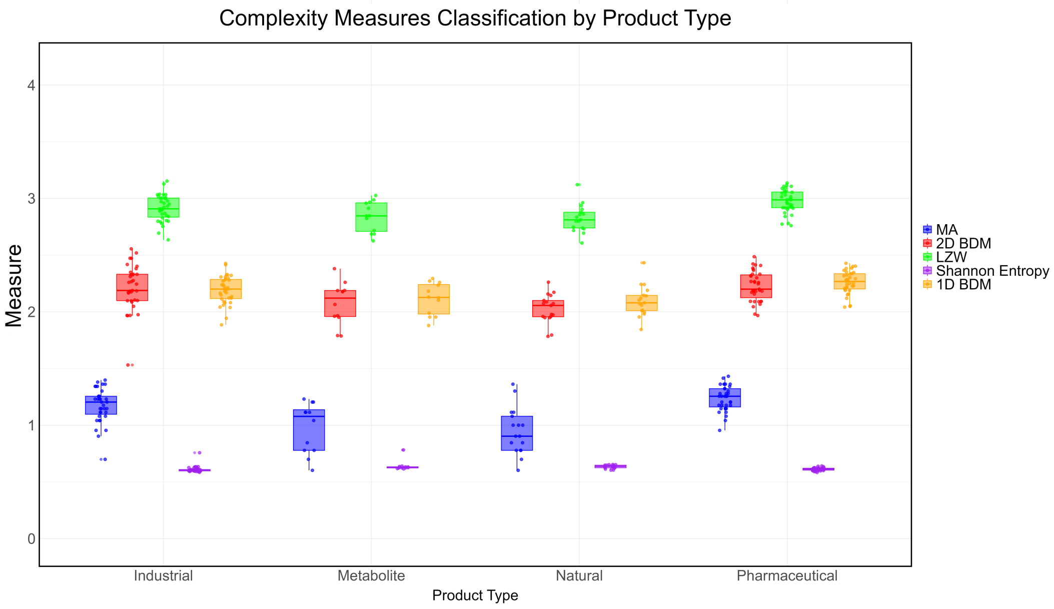


Supplementary Figure 4: Classification of molecular complexity by multiple complexity indexes originally used to create the chemical space for the mass spectroscopy (MS) profiles (log-scale). All measures other than MA applied to bond molecular distance matrices some of which outperform MA and their mass spectra at distinguishing organic from non-organic molecules found in the MS dataset of the MA paper [26], as demonstrated by greater separation and smaller variance results across the different complexity measures among the molecular subgroups. MA does not display any particular advantage when compared against proper control experiments, and performs similarly to the simplest of the statistical algorithms applied to all the tested data representations including molecular distance matrices (as shown here for all measures but MA) or the mass spectral data provided by the authors of Assembly Theory (shown on the plot from the authors' results that could not be fully reproduced due to lack of data made available in [28] but we took at face value) for comparison purposes.

ago when computer compression algorithms where designed based upon the same modular statistical principle of repetition and modular counting re-introduced by MA. Nor were the MA indices able to show any particular advantage over indices that are non-computable but capable of being approximated from above and based on resource-bounded variants of algorithmic complexity (such as BDM [41, 44]), which the authors of MA disqualify a priori [28] without any evidence or control experiments, on account of their

semi-computable nature (where 'semi' means they can be approximated using various methods, as is the case, for example, with protein folding).

| **Statistic** | **MA** | **LZW** | **1D-BDM** |
|---|---|---|---|
| Pearson Correlation (R) | 0.897 | 0.902 | 0.905 |
| 99% confidence interval | 0.832 - 0.938 | 0.84 - 0.941 | 0.845 - 0.943 |
| R squared | 0.805 | 0.815 | 0.820 |

Supplementary Table 2: Table of Pearson correlation values corresponding to Fig. 1. LZW and BDM are given in bits, meaning the length of a compressed description of the object, including the number of steps. Both LZW and BDM generate better separating statistics on InChI codes than MA without any adaptions or modifications.

The correlation analysis suggests a stronger positive linear relationship between MW and measures from algorithmic information dynamics, such as BDM and LZW, in contrast to that between MW and MA. As such, other indices on other chemically derived data representation can be better predictors of increasing molecular complexity in the MS signatures classification as reported before in [35].

MA and Shannon Entropy had a similar statistical significance in classifying the mass spectroscopy (MS) data into their four distinct categories, with t-values of 15.96 and 20.96, respectively at df = 100. The Kolmogorov-Smirnov distances were 0.828 and 1, respectively.

The suggestion of Assembly Theory was that MA can predict living *vs.* nonliving molecules tested on a cherry-picked small subset of biological extracts, between abiotic factors, and inorganic (dead), as shown in [28, Figure 4]. We repeated the experiment using the binarised MS2 spectra peaks matrices provided in the source data in [26]. 18 of the extracts and molecular MS2 spectra were obtained, ignoring the blinded samples shown in [28, Figure 4]. Our reproduced findings on their [28, Figure 4], are shown in our Sup. Fig. 5. By including the 114 molecules from [28, Figure 3] with the 18 molecules of [28, Figure 4], we performed correlation analysis on all 132 signatures, with 5 categories: small molecules, peptides, abiotic, dead (inorganic, such as coal and quartz) and biological extracts (which includes yeast, E.coli, etc.). The Pearson correlation was strongest between 1D-BDM and the category (R= 0.951), followed by 1D-RLE and 1D-Huffman having a near-identical Pearson correlation of R= 0.843 and R= 0.842, respectively. MA has the poorest correlation with the categories, with a correlation of R= 0.448. All Pearson scores were statistically significant (P<0.0001). The

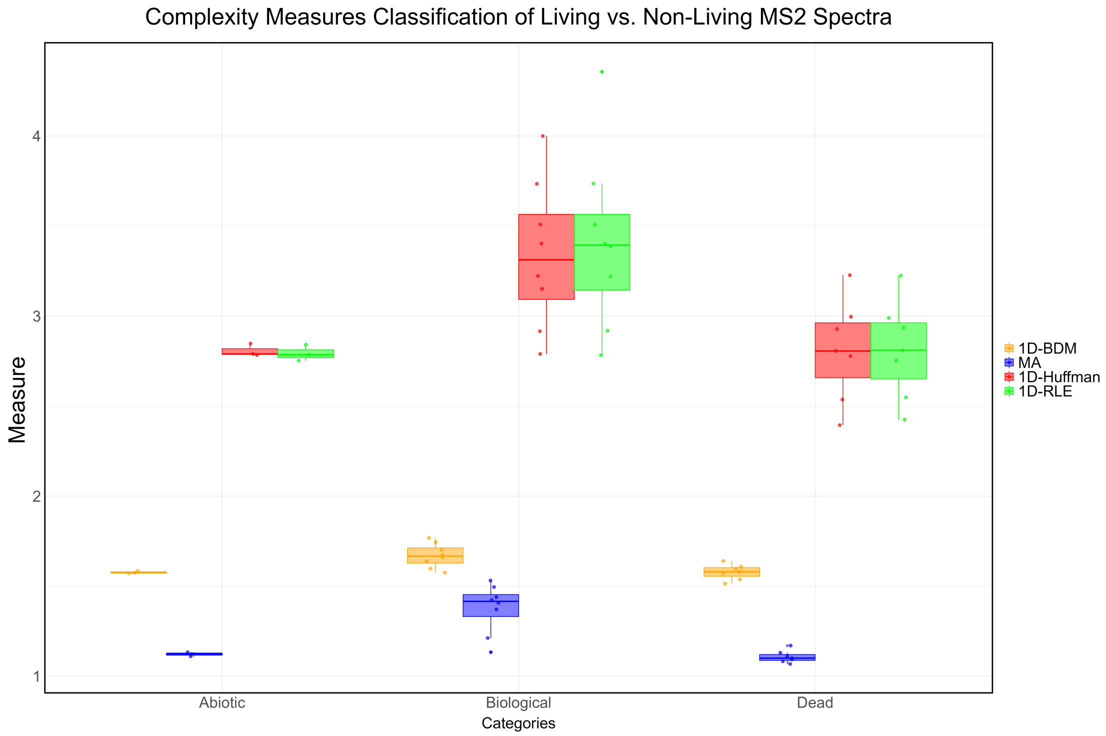


Supplementary Figure 5: Analysis of organic versus non-organic molecules from mass spectral data by multiple complexity indexes: The strongest positive correlation was identified between MA and 1D-RLE coding (R= 0.9), which is one of the most basic coding schemes and among the most similar to the intended definition of MA, as being capable of 'counting copies' in 18 extracts for which the mass spectra was available. Other coding algorithms, including LZ and Huffman coding (R = 0.896), also show a strong positive correlation with MA. As seen, the compression values of both 1D-RLE and 1D-Huffman coding show overlapping and nearly identical medians (horizontal line at centre) and ranges on the whisker plot. The analysis further confirms our previous findings, in the similarity in performance between MA and popular statistical compression measures (whose purpose is also to count identical statistical copies) as we make the case that MA is one (and the same as LZ) in classifying living *vs.* non-living mass spectra signatures.

results for all 132 molecules are shown in Sup. Table 3. Therefore, our findings collectively conclude that when considering both the mass spectrometry signatures of [28, Figures 2B, 3, and 4], together, the coding indexes systematically outperform MA index, as a discriminant of living *vs.* non-living systems.

| Statistics | 1D-BDM | 1D-RLE | 1D-Huffman | MA |
|---|---|---|---|---|
| Pearson Correlation (R) | 0.95 | 0.84 | 0.84 | 0.45 |
| 99% confidence interval | 0.96-0.93 | 0.88-0.78 | 0.88-0.78 | 0.57-0.3 |
| R squared | 0.904 | 0.711 | 0.709 | 0.201 |

Supplementary Table 3: Table of Pearson Correlation values of MA and complexity measures across all 132 molecules, including the biological extracts from [28, Figure 4]. BDM, RLE, and Huffman are given in log-normalized bits. As shown, BDM and the compression algorithms generate better statistics than MA.

There is a significant level of variance in the MA scores of these biological mixtures and extracts, as indicated in [28, Figure 4]. Further, given that the MA or complexity of the biological extracts shown in [28, Figure 4] are mixtures derived from the small molecules and peptides in the MA chemical space constructed from [28, Figure 3] data, and by virtue of other coding indexes outperforming their chemical MA space classification, we can conclude that MA theory is sub-optimal and a limited subset of compression measures provided within the algorithmic complexity framework.

This section completes the control experiments missing in [26, 28, 31] where it is claimed novelty at ‘counting copies’ as a measure or its application to a special type of data that is not needed to produce the same results (from any tested data).

| Measure | Kruskal-Wallis p-value | ANOVA p-value |
|---|---|---|
| 1D-BDM | 0.02 | 0.01 |
| MA | 0.00 | 0.00 |
| 1D-Huffman | 0.03 | 0.01 |
| 1D-RLE | 0.03 | 0.01 |

Supplementary Table 4: The Kruskal-Wallis and ANOVA statistical significance test on Mass Spectrometry Data. Validation by Kruskal-Wallis and ANOVA tests along with one-tailed t-tests of the MS2 data analysis shown in Figure 2 in our main article (i.e., complexity analysis on Dead, Inorganic, and Biological samples) or Supplementary Figure 5 can provide more robust and credible significance testing (p-values) by examining the data from multiple statistical perspectives. Kruskal-Wallis is a non-parametric alternative to ANOVA, and using both parametric and non-parametric tests can help validate the findings and rule out potential issues with normality assumptions. Both test results were computed using the `scipy.stats` package in Python.

# References


[1] Felipe S. Abrahão. The "paradox" of computability and a recursive relative version of the Busy Beaver function. In Cristian Calude and Mark Burgin, editors, *Information and Complexity*, chapter 1, pages 3–15. World Scientific Publishing, Singapore, 1 edition, 2016. ISBN 978-9813109025. doi: 10.1142/9789813109032_0001.

[2] Felipe S. Abrahão, Klaus Wehmuth, and Artur Ziviani. Algorithmic networks: Central time to trigger expected emergent open-endedness. *Theoretical Computer Science*, 785:83–116, sep 2019. ISSN 03043975. doi: 10.1016/j.tcs.2019.03.008.

[3] Felipe S. Abrahão, Klaus Wehmuth, Hector Zenil, and Artur Ziviani. An Algorithmic Information Distortion in Multidimensional Networks. In Rosa M. Benito, Chantal Cherifi, Hocine Cherifi, Esteban Moro, Luis Mateus Rocha, and Marta Sales-Pardo, editors, *Complex Networks & Their Applications IX*, volume 944 of *Studies in Computational Intelligence*, pages 520–531, Cham, 2021. Springer International Publishing. ISBN 978-3-030-65351-4. doi: 10.1007/978-3-030-65351-4_42.

[4] Felipe S. Abrahão, Klaus Wehmuth, Hector Zenil, and Artur Ziviani. Algorithmic information distortions in node-aligned and node-unaligned

multidimensional networks. *Entropy*, 23(7), 2021. ISSN 1099-4300. doi: 10.3390/e23070835.

[5] Felipe S. Abrahão, Hector Zenil, Fabio Porto, Michael Winter, Klaus Wehmuth, and Itala M. L. D'Ottaviano. A simplicity bubble problem in formal-theoretic learning systems. *arXiv Preprints*, 2023. URL `http://arxiv.org/abs/2112.12275v2`.

[6] Felipe S. Abrahão, Santiago Hernández-Orozco, Narsis A. Kiani, Jesper Tegnér, and Hector Zenil. Assembly theory is an approximation to algorithmic complexity based on lz compression that does not explain selection or evolution. *arXiv:2403.06629 [cs.IT]*, 2024. URL `https://arxiv.org/abs/2403.06629`.

[7] Felipe S. Abrahão and Hector Zenil. Emergence and algorithmic information dynamics of systems and observers. *Philosophical Transactions of the Royal Society A: Mathematical, Physical and Engineering Sciences*, 380(2227), 2022. ISSN 1364-503X. doi: 10.1098/rsta.2020.0429.

[8] Eric Allender, Michal Koucký, Detlef Ronneburger, and Sambuddha Roy. The pervasive reach of resource-bounded Kolmogorov complexity in computational complexity theory. *Journal of Computer and System Sciences*, 77(1):14–40, 2011. ISSN 00220000. doi: 10.1016/j.jcss.2010.06.004.

[9] Verónica Becher and Santiago Figueira. An example of a computable absolutely normal number. *Theoretical Computer Science*, 270(1-2):947–958, jan 2002. ISSN 03043975. doi: 10.1016/S0304-3975(01)00170-0.

[10] Mark Burgin. *Theory of Information: Fundamentality, Diversity and Unification.* World Scientific Publishing, 2009. ISBN 978-981-283-548-2 978-981-283-549-9. doi: 10.1142/7048.

[11] Cristian S. Calude. *Information and Randomness: An algorithmic perspective.* Springer-Verlag, 2 edition, 2002. ISBN 3540434666.

[12] Gregory Chaitin. *Algorithmic Information Theory.* Cambridge University Press, 3 edition, 2004. ISBN 0521616042.

[13] R. Cilibrasi and P.M.B. Vitanyi. Clustering by Compression. *IEEE Transactions on Information Theory*, 51(4):1523–1545, 2005. ISSN 0018-9448. doi: 10.1109/TIT.2005.844059.

[14] Thomas M. Cover and Joy A. Thomas. *Elements of Information Theory.* John Wiley & Sons, Inc., Hoboken, NJ, USA, sep 2005. ISBN 9780471241959. doi: 10.1002/047174882X.

[15] Leroy Cronin. On the Salient Limitations of 'On the Salient Limitations of the Methods of Assembly Theory and their Classification of Molecular Biosignatures'. *arXiv Preprints*, arXiv:2403.12452, 2024.

[16] Jean Paul Delahaye and Hector Zenil. Numerical evaluation of algorithmic complexity for short strings: A glance into the innermost structure of randomness. *Applied Mathematics and Computation*, 219(1):63–77, sep 2012. ISSN 00963003. doi: 10.1016/j.amc.2011.10.006.

[17] Rod Downey and Denis R. Hirschfeldt. Algorithmic randomness. *Communications of the ACM*, 62(5):70–80, apr 2019. ISSN 00010782. doi: 10.1145/3319408.

[18] Rodney G. Downey and Denis R. Hirschfeldt. *Algorithmic Randomness and Complexity.* Theory and Applications of Computability. Springer New York, New York, NY, 2010. ISBN 978-0-387-95567-4. doi: 10.1007/978-0-387-68441-3.

[19] Nicolas Gauvrit, Hector Zenil, Fernando Soler-Toscano, Jean-Paul Delahaye, and Peter Brugger. Human behavioral complexity peaks at age 25. *PLOS Computational Biology*, 13(4):e1005408, 2017. ISSN 1553-7358. doi: 10.1371/journal.pcbi.1005408.

[20] Ian Goodfellow, Yoshua Bengio, and Aaron Courville. *Deep Learning.* MIT Press, 2016. `http://www.deeplearningbook.org`.

[21] Santiago Hernández-Orozco, Narsis A. Kiani, and Hector Zenil. Algorithmically probable mutations reproduce aspects of evolution, such as convergence rate, genetic memory and modularity. *Royal Society Open Science*, 5(8):180399, 2018. doi: 10.1098/rsos.180399.

[22] Wilfrid Hodges. *Model Theory.* Cambridge University Press, 1 edition, March 1993. ISBN 978-0-521-30442-9 978-0-521-06636-5 978-0-511-55157-4. doi: 10.1017/CBO9780511551574.

[23] David Huffman. A method for the construction of minimum-redundancy codes. *Proceedings of the IRE*, 40(9):1098–1101, 1952. doi: 10.1109/jrproc.1952.273898.

[24] Ming Li and Paul Vitányi. *An Introduction to Kolmogorov Complexity and Its Applications.* Texts in Computer Science. Springer, Cham, 4 edition, 2019. ISBN 978-3-030-11298-1. doi: 10.1007/978-3-030-11298-1.

[25] Seth Lloyd and Heinz Pagels. Complexity as thermodynamic depth. *Annals of Physics*, 188(1):186–213, 1988. doi: 10.1016/0003-4916(88)90094-2.

[26] Stuart M. Marshall, Alastair R. G. Murray, and Leroy Cronin. A probabilistic framework for identifying biosignatures using pathway complexity. *Philosophical Transactions of the Royal Society A: Mathematical, Physical and Engineering Sciences*, 375(2109):20160342, 2017. doi: 10.1098/rsta.2016.0342.

[27] Stuart M. Marshall, Douglas Moore, Alastair R. G. Murray, Sara I. Walker, and Leroy Cronin. Quantifying the pathways to life using assembly spaces. *arXiv Preprints*, 2019. URL `http://arxiv.org/abs/1907.04649`.

[28] Stuart M. Marshall, Cole Mathis, Emma Carrick, Graham Keenan, Geoffrey J. T. Cooper, Heather Graham, Matthew Craven, Piotr S. Gromski, Douglas G. Moore, Sara. I. Walker, and et al. Identifying molecules as biosignatures with assembly theory and mass spectrometry. *Nature Communications*, 12(1), 2021. doi: 10.1038/s41467-021-23258-x.

[29] Turlough Neary and Damien Woods. P-completeness of Cellular Automaton Rule 110. In David Hutchison, Takeo Kanade, Josef Kittler, Jon M. Kleinberg, Friedemann Mattern, John C. Mitchell, Moni Naor, Oscar Nierstrasz, C. Pandu Rangan, Bernhard Steffen, Madhu Sudan, Demetri Terzopoulos, Dough Tygar, Moshe Y. Vardi, Gerhard Weikum, Michele Bugliesi, Bart Preneel, Vladimiro Sassone, and Ingo Wegener, editors, *Automata, Languages and Programming*, volume 4051, pages 132–143. Springer Berlin Heidelberg, Berlin, Heidelberg, 2006. ISBN 978-3-540-35904-3. doi: 10.1007/11786986_13. Series Title: Lecture Notes in Computer Science.

[30] A.H. Robinson and C. Cherry. Results of a prototype television bandwidth compression scheme. *Proceedings of the IEEE*, 55(3):356–364, 1967. ISSN 0018-9219. doi: 10.1109/PROC.1967.5493.

[31] Abhishek Sharma, Dániel Czégel, Michael Lachmann, Christopher P. Kempes, Sara I. Walker, and Leroy Cronin. Assembly theory explains

and quantifies selection and evolution. *Nature*, 622(7982):321–328, 2023. ISSN 0028-0836. doi: 10.1038/s41586-023-06600-9.

[32] Fernando Soler-Toscano, Hector Zenil, Jean-Paul Delahaye, and Nicolas Gauvrit. Calculating kolmogorov complexity from the output frequency distributions of small turing machines. *PLoS ONE*, 9(5), 2014. doi: 10.1371/journal.pone.0096223.

[33] Sara Imari Walker and Paul C. W. Davies. The Algorithmic Origins of Life. *Journal of The Royal Society Interface*, 10(79), 2012. ISSN 1742-5689. doi: 10.1098/rsif.2012.0869.

[34] Stephen Wolfram. *A new kind of science*. Wolfram Media, Champaign, IL, 2002. ISBN 978-1-57955-008-0.

[35] H. Zenil, N.A. Kiani, and J. Tegnér. Algorithmic Complexity and Reprogrammability of Chemical Structure Networks. *Parallel Processing Letters*, 28, 2018. doi: 10.1142/s0129626418500056.

[36] Hector Zenil. A review of methods for estimating algorithmic complexity: options, challenges, and new directions. *Entropy*, 22(6):612, 2020. ISSN 1099-4300. doi: 10.3390/e22060612. URL `https://www.mdpi.com/1099-4300/22/6/612`.

[37] Hector Zenil and Peter Minary. Training-free measures based on algorithmic probability identify high nucleosome occupancy in DNA sequences. *Nucleic Acids Research*, 47(20):e129–e129, 2019. ISSN 0305-1048. doi: 10.1093/nar/gkz750.

[38] Hector Zenil and Elena Villarreal-Zapata. Asymptotic Behavior And Ratios of Complexity In Cellular Automata. *International Journal of Bifurcation and Chaos*, 23(09):1350159, 2013. ISSN 0218-1274, 1793-6551. doi: 10.1142/S0218127413501599.

[39] Hector Zenil, Jean-Paul Delahaye, and Cédric Gaucherel. Image characterization and classification by physical complexity. *Complexity*, 17(3): 26–42, 2011. doi: 10.1002/cplx.20388.

[40] Hector Zenil, Carlos Gershenson, James Marshall, and David Rosenblueth. Life as Thermodynamic Evidence of Algorithmic Structure in Natural Environments. *Entropy*, 14(11):2173–2191, 2012. ISSN 1099-4300. doi: 10.3390/e14112173.

[41] Hector Zenil, Fernando Soler-Toscano, Jean-Paul Delahaye, and Nicolas Gauvrit. Two-dimensional Kolmogorov complexity and an empirical validation of the Coding theorem method by compressibility. *PeerJ Computer Science*, 1, September 2015. doi: 10.7717/peerj-cs.23.

[42] Hector Zenil, Narsis A. Kiani, and Jesper Tegnér. Low-algorithmic-complexity entropy-deceiving graphs. *Physical Review E*, 96(1), 2017. doi: 10.1103/physreve.96.012308.

[43] Hector Zenil, Santiago Hernández-Orozco, Narsis Kiani, Fernando Soler-Toscano, Antonio Rueda-Toicen, and Jesper Tegnér. A Decomposition Method for Global Evaluation of Shannon Entropy and Local Estimations of Algorithmic Complexity. *Entropy*, 20(8):605, aug 2018. ISSN 1099-4300. doi: 10.3390/e20080605. URL http://www.mdpi.com/1099-4300/20/8/605.

[44] Hector Zenil, Narsis A. Kiani, Francesco Marabita, Yue Deng, Szabolcs Elias, Angelika Schmidt, Gordon Ball, and Jesper Tegnér. An algorithmic information calculus for causal discovery and reprogramming systems. *iScience*, 19:1160–1172, 2019. doi: 10.1016/j.isci.2019.07.043.

[45] Hector Zenil, Narsis A. Kiani, Allan A. Zea, and Jesper Tegnér. Causal deconvolution by algorithmic generative models. *Nature Machine Intelligence*, 1(1):58–66, jan 2019. ISSN 2522-5839. doi: 10.1038/s42256-018-0005-0. URL http://www.nature.com/articles/s42256-018-0005-0.

[46] Hector Zenil, Narsis Kiani, Felipe S. Abrahão, and Jesper Tegnér. Algorithmic Information Dynamics. *Scholarpedia Journal*, 15(7):53143, 2020. ISSN 1941-6016. doi: 10.4249/scholarpedia.53143.

[47] Hector Zenil, Narsis A. Kiani, and Jesper Tegnér. *Algorithmic Information Dynamics: A Computational Approach to Causality with Applications to Living Systems.* Cambridge University Press, 1 edition, May 2023. ISBN 978-1-108-59661-9 978-1-108-49766-4. doi: 10.1017/9781108596619. URL https://www.cambridge.org/core/product/identifier/9781108596619/type/book.
